# Stretchable Triboelectric Nanogenerator Based on Liquid Metal with Varying Phases

**DOI:** 10.1002/advs.202405792

**Published:** 2024-08-13

**Authors:** Li Yang, Langang Guo, Zihan Wang, Chuizhou Meng, Jinrong Wu, Xue Chen, Abdullah Abu Musa, Xiaoqi Jiang, Huanyu Cheng

**Affiliations:** ^1^ State Key Laboratory of Reliability and Intelligence of Electrical Equipment School of Health Sciences and Biomedical Engineering Hebei University of Technology Tianjin 300130 China; ^2^ State Key Laboratory for Reliability and Intelligence of Electrical Equipment Hebei Key Laboratory of Smart Sensing and Human‐Robot Interaction School of Mechanical Engineering Hebei University of Technology Tianjin 300401 China; ^3^ State Key Laboratory of Polymer Material Engineering College of Polymer Science and Engineering Sichuan University Chengdu 610065 China; ^4^ State Key Laboratory of Reliability and Intelligence of Electrical Equipment Key Laboratory of Bioelectromagnetics and Neuroengineering of Hebei Province School of Electrical Engineering Hebei University of Technology Tianjin 300130 China; ^5^ Department of Engineering Science and Mechanics The Pennsylvania State University University Park 16802 USA

**Keywords:** liquid metal, multi‐substrate electrode, stretchable, triboelectric nanogenerators

## Abstract

Stretchable triboelectric nanogenerators (TENGs) represent a new class of energy‐harvesting devices for powering wearable devices. However, most of them are associated with poor stretchability, low stability, and limited substrate material choices. This work presents the design and demonstration of highly stretchable and stable TENGs based on liquid metalel ectrodes with different phases. The conductive and fluidic properties of eutectic gallium‐indium (EGaIn) in the serpentine microfluidic channel ensure the robust performance of the EGaIn‐based TENG upon stretching over several hundred percent. The bi‐phasic EGaIn (bGaIn) from oxidation lowers surface tension and increases adhesion for printing on diverse substrates with high output performance parameters. The optimization of the electrode shapes in the bGaIn‐based TENGs can reduce the device footprint and weight, while enhancing stretchability. The applications of the EGaIn‐ and bGaIn‐based TENG include smart elastic bands for human movement monitoring and smart carpets with integrated data transmission/processing modules for headcount monitoring/control. Combining the concept of origami in the paper‐based bGaIn TENG can reduce the device footprint to improve output performance per unit area. The integration of bGaIn‐TENG on a self‐healing polymer substrate with corrosion resistance against acidic and alkaline solutions further facilitates its use in various challenging and extreme environments.

## Graphical abstract

This work presents stretchable and stable triboelectric nanogenerators (TENGs) based on liquid metal with different crystalline phases. The eutectic gallium‐indium(EGaIn)‐based TENG can be stretched over 800%, whereas the bi‐phasic‐EGaIn (bGaIn) lowers surface tension and increases adhesion for printing on diverse substrates. The optimization of electrode shapes in the bGaIn‐based TENGs reduces the device footprint and weight while providing stretchable properties.

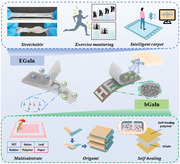

## 1 Introduction

The rapid development of smart electronic products such as wearable devices, biomedical monitors, and wireless sensor networks poses increased demands for sustainable power supplies.^[^ ^1^, ^2^, ^3^ ^]^ However, conventional power supplies such as batteries exhibit limited storage capacity, flexibility, and lifespan, and often cause environmental concerns.^[^ ^4^, ^5^, ^6^, ^7^, ^8^ ^]^ To harvest the clean mechanical energy from the ambient environment (e.g., human motion, wind vibration, and water flow), triboelectric nanogenerators (TENGs) recently developed have been explored to convert low‐frequency mechanical energy into electrical energy based on triboelectrification and electrostatic induction.^[^ ^9^ ^]^ This class of energy harvesting modules features low fabrication cost, high conversion efficiency, easily accessible materials, and environmental friendliness.^[^ ^10^, ^11^, ^12^, ^13^ ^]^ Despite a wide range of triboelectric materials,^[^ ^14^, ^15^, ^16^, ^17^ ^]^ the electrode materials are still limited to metals and metallic materials that often lack stretchability and easy integration on diverse substrates. For instance, the traditional conductive materials in TENG fabrication include rigid metals (copper and aluminum),^[^ ^18^, ^19^, ^20^, ^21^ ^]^ nanocomposites (with embedded carbon nanotubes, graphene, and silver nanowires),^[^ ^22^, ^23^, ^24^, ^25^ ^]^ ionic solutions,^[^ ^26^, ^27^, ^28^, ^29^ ^]^ and cross‐linked gels.^[^ ^30^, ^31^, ^32^, ^33^ ^]^ However, they suffer from high Young's modulus, complex preparation processes, and poor stability, presenting challenges in flexible/stretchable TENGs.

As a promising alternative to flexible/stretchable electrodes, the liquid metal (LM) based on eutectic gallium‐indium (EGaIn) shows low melting point, high fluidity, high conductivity, and low toxicity.^[^ ^34^, ^35^, ^36^, ^37^ ^]^ However, the oxidization of EGaIn results in a thin surface oxide layer (≈3 nm). The high surface tension (≈600 mN m^−1^) and poor adhesion^[^ ^38^ ^]^ of the surface oxide present challenges to depositing on diverse smooth surfaces.^[^ ^39^ ^]^ Although microfluidic channels can be used for the fabrication of TENGs,^[^ ^40^, ^41^ ^]^ they often exhibit limited stretchability and stability and can only be used on certain substrate materials. While composites,^[^ ^42^ ^]^ ultrasonic‐assisted sintering,^[^ ^43^ ^]^ and other processes^[^ ^44^ ^]^ have been exploited to improve the wettability between LM and substrates, they tend to have complicated preparation processes and single substrate adaptation. Therefore, there is still a critical demand to design and fabricate bi‐phasic EGaIn(bGaIn)‐based stretchable TENG on diverse substrates in different operating environments.

This work presents an effective fabrication method to respectively prepare stretchable and substrate‐adaptable TENGs by using liquid metals with different crystalline phases as electrode materials. The highly stretchable and tribo‐negative silicone rubber with patterned serpentine microfluidic structure inside^[^ ^45^, ^46^ ^]^ encapsulates the EGaIn to provide stretchable TENG with enhanced stability and stretchability of up to 876%, allowing for integration with elastic bands for self‐powered sensing of large human motions.^[^ ^47^ ^]^ Oxidation of EGaIn further results in biphasic EGaIn (bGaIn) with reduced surface tension and improved adhesion for direct patterning on diverse substrates without limitations on the thickness. The resulting bGaIn‐based TENG on various substrates with tunable properties features reduced thickness and weight for applications that require self‐healing or in acidic and alkaline environments. The applications of the resulting TENGs also include smart carpets for people stream monitoring and controlling, wind/rain monitoring, gesture recognition, and origami paper‐based TENG for improved output performance per unit area.

## 2 Results

### 2.1 Preparation of TENGs Based on Liquid Metal Electrodes

**Figure 1:**
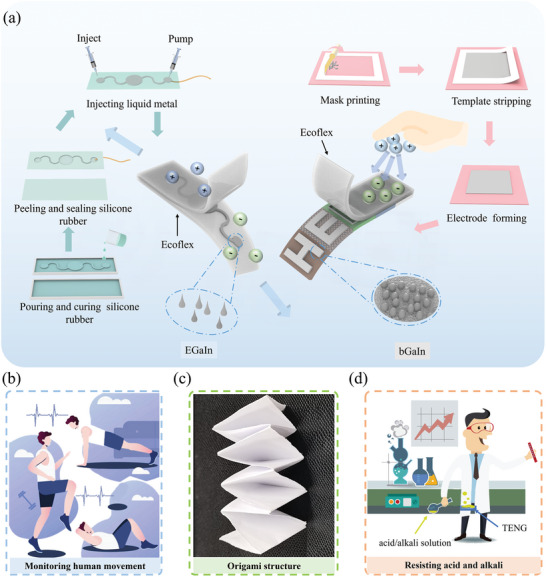
Schematic diagram showing a) the design and fabrication of TENG based on liquid metal electrodes, with applications b) to monitor human movement, c) in space‐limited regions by exploring origami structures, and d) in acidic and alkaline environments.

The liquid metal with different crystal phases allows the design and fabrication of the TENGs on diverse substrates with facilely tunable performance parameters. The 3D printed serpentine microfluidic structure with commonly used liquid metal EGaIn injected (**Figure** **1a‐left**) provides stretchable TENGs with enhanced stretchability and increased stability upon stretching (Figure S1, Supporting Information). However, the thick microfluidic structure results in a large device weight. In contrast, the use of the directly patterned bGaIn on diverse thin substrates (Figures S2 and S3, Supporting Information) with kirigami designs (Figure S4, Supporting Information) creates lightweight yet high‐performance TENGs (Figure 1a‐right). The resulting EGaIn‐based stretchable TENG can be used as a self‐powered sensor to monitor the human body's movement status (Figure 1b). The applications of the substrate‐adaptable bGaIn‐based TENG include a smart carpet for people monitoring and controlling, soft monitors for wind speed and rainfall in natural environments, and human‐machine interfaces for gesture recognition. For the applications with constraints in device footprint, the TENG can be combined with the concept of origami to result in the device in folded configurations with improved output characteristics per unit area (Figure 1c). The exploration of thin substrates with tailored properties such as acid‐ and alkali‐tolerant polymers further opens up the application opportunities to use TENGs in acidic and alkaline environments during rescue (Figure 1d).

### 2.2 Characterization of the Liquid Metal before and after Oxidation

**Figure 2:**
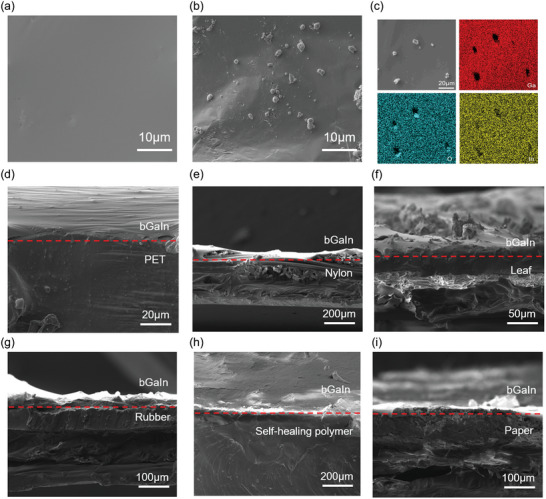
SEM images of EGaIn in a) liquid phase state and b) biphasic state after oxidation (i.e., bGaIn). c) Energy dispersive spectrogram of bGaIn, with higher oxygen and gallium concentration in the region of solid particles. SEM images showing cross sections of bGaIn on d) PET, e) nylon, f) leaf, g) rubber, h) self‐healing polymer, and i) paper.

Different from the EGaIn in a liquid phase, the biphasic bGaIn consists of a solid phase partially embedded and tightly wrapped by the liquid phase (**Figure** **2**a,b). The energy dispersive spectrum of bGaIn (Figure 2c) shows that the concentration of oxygen and gallium in the solid particle region is higher than that in the liquid region (with vanishing In), indicating the oxidation of Ga into Ga_2_O_3_ particles.^[^ ^48^ ^]^ The XPS results reveal strong Ga (2p, 3p, 3d) peaks and O (1s) peaks, indefinite C (1s) peaks, and weak In (3d) peaks for both EGaIn and bGaIn. However, the binding energy of Ga2p1/Ga2p3 shifts from 1142.6/1115.8 eV for EGaIn to 1145/1118 eV for bGaIn (Figures S5 and S6, Supporting Information), which may be attributed to the increased Ga_2_O_3_ content.^[^ ^49^, ^50^ ^]^ No obvious peaks in the XRD patterns of EGaIn and bGaIn indicate the amorphous state of Ga_2_O_3_ (Figure S7, Supporting Information), because the fabrication temperature of bGaIn is far lower than the crystallization temperature (≈350 °C) of the oxidation product Ga_2_O_3_. The peeling experiments under the same conditions (Figure S8, Supporting Information) reveal the differences in the adhesion between LM and substrates, with adhesion enhanced by the crosslinked structural form of ionic networks in polymers. The solid‐phase oxide particles in bGaIn reduce the surface tension of bGaIn through rigid contacts and interactions between liquid bridges, which reduces mobility and improves stability.^[^ ^51^ ^]^ The results of the contact angle test (Figure S9, Supporting Information) show different contact states of bGaIn on various substrates (e.g., the contact angles of 138.8/57.4° for EGaIn/bGaIn on polyethyleneterephthalate (PET)61 substrates are reduced to 98.1/16.3° on paper substrates), which may be related to the substrate adhesion difference. The process of printing bGaIn onto different substrates under external forces forms a composite consisting of the old/new oxide and bare liquid metal at the bGaIn/substrate interface, which results in intimate contact between the new oxide and substrate surface.^[^ ^52^ ^]^ The formed gaps between bGaIn and different substrates in the SEM images (Figure 2d–i) reveal the difference in adhesion and the bonding effect. The smooth surface of the PET substrate has poor adhesion to bGaIn.^[^ ^53^ ^]^ The rough surface of nylon fabric (Nylon 66) exhibits enhanced adhesion, but the smoothness of the nylon wire material itself affected the adhesion of bGaIn.^[^ ^54^ ^]^ The micro bumps on the surface of plant leaves also provide enhanced adhesion, but the superhydrophobic epidermal waxes on the surface reduce the printing accuracy of bGaIn.^[^ ^55^ ^]^ After the rupture of bGaIn, the contact interface formed between the oxide shell and the bumps of nitrile rubber provides enhanced adhesion and printing of bGaIn.^[^ ^56^ ^]^ The high adhesion provided by the crosslinked structural form of ionic networks in the self‐healing polymer can also facilitate the printing of bGaIn. The adhesion of the paper‐based substrate is not optimal, but the interlaced fiber structure allows the liquid‐phase bGaIn to penetrate for enhanced adhesion.^[^ ^57^ ^]^

### 2.3 Output Characteristics of the Stretchable TENG in the Single Electrode Mode

**Figure 3:**
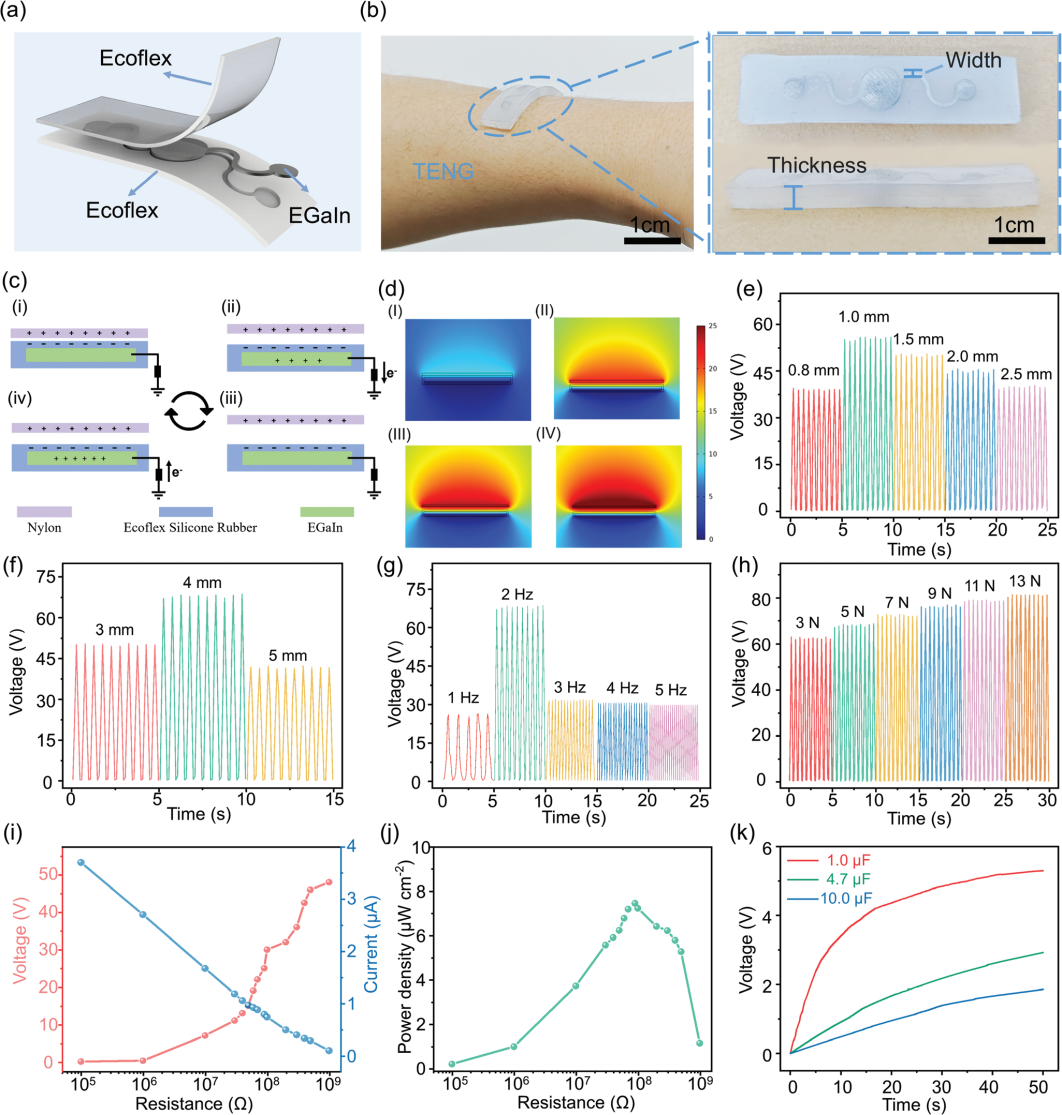
The working mechanism and output performance of stretchable TENG in single electrode mode. a) Structural schematic and b) optical image of the liquid metal TENG. c) Working principle and d) finite element simulation of the LM‐based TENG over a contact‐separation cycle. Comparison of TENG output voltages for different e) channel widths (thickness of 3 mm, 5 N at 2 Hz) and f) thicknesses (channel width of 1 mm, 5 N at 2 Hz), or g) frequencies (for 5 N) and h) forces (at 2 Hz) (both with channel width of 1 mm and thickness of 3 mm). i) Output voltage and current and j) peak power density as a function of the external load resistance from 10^5^ to 10^9^ Ω. k) Demonstration of the TENG to charge commercial capacitors of 1, 4.7, and 10 µF.

By exploring the silicone elastomer with different widths and thicknesses in the embedded serpentine microfluidics (**Figure** **3**a), the stretchable TENG can be tightly attached to the skin (Figure 3b) as a single electrode TENG as the nylon layer (Figure S10, Supporting Information) moves out of the plane (Figure 3c). As the nylon fabric touches the silicone rubber, electrons move from the tribo‐positive nylon surface to the tribo‐negative rubber surface due to the triboelectrification effect^[^ ^58^ ^]^ (Figure 3c‐i) and there is no electron flow in the external circuit due to charge equilibrium. As the nylon fabric moves away from the silicone rubber, the electrons flow from the liquid metal to the ground through the external circuit, generating voltage/current output signals (Figure 3c‐ii). When the nylon fabric moves further away, the charge imbalance in the tribo‐negative layer due to the surface charge transfer is neutralized by the additional positive charges on the electrode, forming an electrostatic balance and giving no current signal in the external circuits (Figure 3c‐iii). Moving the nylon fabric to the silicone rubber reverses the electron flow from the ground to the liquid metal (Figure 3c‐iv), resulting in a negative output signal. The potential distribution under the contact‐separation mode is simulated by COMSOL Multiphysics software (Figure 3d).

Optimization of the device output performance relies on the study of the effect of structural parameters. Changing the channel width from 0.8 to 2.5 mm (for applied load force of 5 N at frequency of 2 Hz, and fixed thickness of 3 mm) shows the best performance with an open‐circuit voltage of 55.8 V, short‐circuit current density of 871.4 µA m^−2^, and transferred charge density of 25.3 µC m^−2^ at a channel width of 1.0 mm (Figure 3e; Figure S11a, Supporting Information). The reduced performance for the width exceeding 1 mm probably results from the fixed volume of EGaIn to fill the microfluidic channel, with the increased width to create air gaps. While a small air gap can promote the generation of induced charges, a large air gap could reduce the contact area between the silicone rubber and EGaIn to affect the accumulation of friction charges and output characteristics.^[^ ^59^, ^60^ ^]^ When the silicone rubber thickness increases from 3 to 5 mm with the channel thickness fixed at 1 mm (Figure 3f; Figure S11b, Supporting Information), the electrical output first increases and then reduces possibly due to the charge retention capacity of the electrification layer. As the channel thickness increases, the enhanced resistance to the internal electric field produces higher surface charge densities,^[^ ^61^ ^]^ but the equivalent capacitance would reduce the charge storage capacity based on the equivalent capacitance model (σ = ε_0_ ε ΔV/d, where ε_0_ and ε are the permittivity of vacuum and the triboelectric layer, ΔV is charging voltage, and d is the thickness of the triboelectric layer).^[^ ^62^ ^]^ As a result, the peak output occurs at a channel thickness of 4 mm to give a voltage of 68.3 V, current density of 957.5 µA m^−2^, and transferred charge density of 35.8 µC m^−2^.

After determining the optimized structural parameters (channel width 1.0 mm, thickness 4 mm), the investigation of the loading frequency from 1 to 5 Hz at 5 N leads to the peak voltage output at 2 Hz (Figure 3g; Figure S11c, Supporting Information). Despite the initial increase in the output with the increasing frequency, the reduced voltage output over 2 Hz likely comes from the mismatch of the resonance frequency of the device,^[^ ^63^ ^]^ which results in poorer contact^[^ ^64^ ^]^ between the friction electrodes and affects the energy conversion efficiency.^[^ ^65^ ^]^ In addition, the buffering effect of the thick device may reduce the energy at high frequencies, leading to reduced charge transfer and output characteristics. Meanwhile, the increase in the external loading force from 3 to 13 N at 2 Hz increases the effective contact area between the friction layers for larger charge transfer^[^ ^66^, ^67^ ^]^ to achieve the output voltage of 81.9 V, current density of 1435.6 µA m^−2^, and transferred charge density of 41.9 µC m^−2^ at 13 N (Figure 3h; Figure S11d, Supporting Information).

As the external load resistance increases from 0.1 MΩ to 1 GΩ, the output voltage increases but the output current decreases (Figure 3i) to result in the peak power density of 7.55 µW cm^−2^ for a load resistance of 90 MΩ (Figure 3j), indicating the same device resistance according to the Jacobi's principle. The alternating current (AC) power generated by the stretchable TENG can be converted to direct current (DC) energy through a bridge rectifier (Figure S12a, Supporting Information) for charging energy storage devices (e.g., capacitor or battery). By using vibration from the exciter or hand tapping (Figure S12b, Supporting Information) at a frequency of 2–3 Hz, the TENG can rapidly charge the capacitors from 1 to 10 µF (Figure 3k) (e.g., up to 3.2 V in 80 s for the 4.7 µF capacitor) (Figure S12c, Supporting Information). The harvested energy can also readily power 430 LED lights, watches, and digital alarm clocks (Figure S13 and Movies [Link], [Link], Supporting Information).

### 2.4 Output Performance of the Stretchable TENG upon Deformation

**Figure 4:**
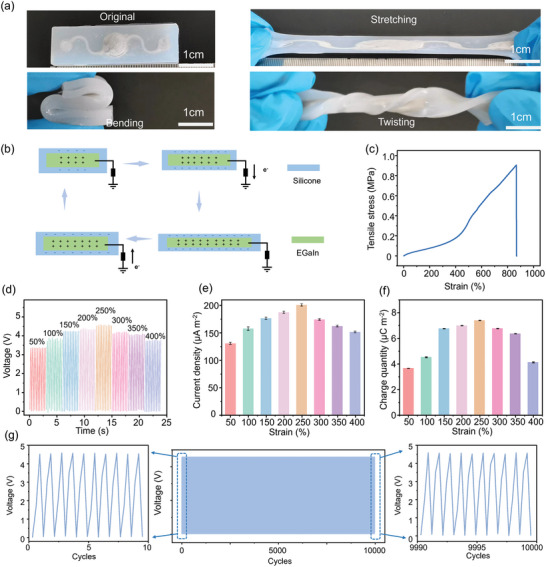
Evaluation of stretchable TENG upon mechanical deformation. a) Optical images of the LM‐based TENG before and after stretching, bending, and twisting. b) The working principle of the TENG upon stretching. c) Stress–strain curve of the LM‐based TENG. d) Voltage output, e) current density, and f) charge density of the LM‐based TENG at different tensile strain levels. g) Stability of the LM‐based TENG over 10000 stretching cycles between 0% and 250%. (TENG with a length of 50 mm, width of 15 mm, thickness of 4 mm, and channel width of 1 mm).

The LM‐based TENG can be bent/folded, twisted, and stretched (**Figure** **4**a). Different from the contact separation between the positive and negative friction layers in the single‐electrode mode, the change of the contact area between the liquid metal and the silicone rubber during stretching results in generated frictional surface charges to neutralize the surface charges (Figure 4b). Therefore, electrons flow between the EGaIn electrode and the earth, creating a continuous AC electrical signal. The stress‐strain curve of the stretchable TENG (Figure 4c) indicates a maximum stretchability of 867%, which has advantage compared with those reported previously (Table S1, Supporting Information). In addition, we have discussed the methods used in similar work to enhance the tensile properties of stretchable TENG as an opportunity to further enhance the performance in future studies. By using a flexible electronic tester (Figure S14 and Movie S4, Supporting Information) to apply the tensile strain from 0 to 400%, the output performance of the stretchable TENG first increases and then decreases with a peak voltage of 4.6 V, current density of 200.1 µA m^−2^, and transferred charge density of 7.4 µC m^−2^ at 250% stretching (Figure 4d–f). The changing trend with increasing strain results from the combined effects^[^ ^68^ ^]^ of changed contact area (first increasing and then decreasing), reduced friction layer thickness from Poisson's effect, enhanced electrostatic induction, and increased electrode resistance (Figure S15, Supporting Information). The stretchable TENG also exhibits high stability over 10 000 cycles between 0% and 250% (Figure 4g; Figure S16, Supporting Information).

### 2.5 Output Characteristics of the bGaIn‐Based TENG on Diverse Substrates

**Figure 5:**
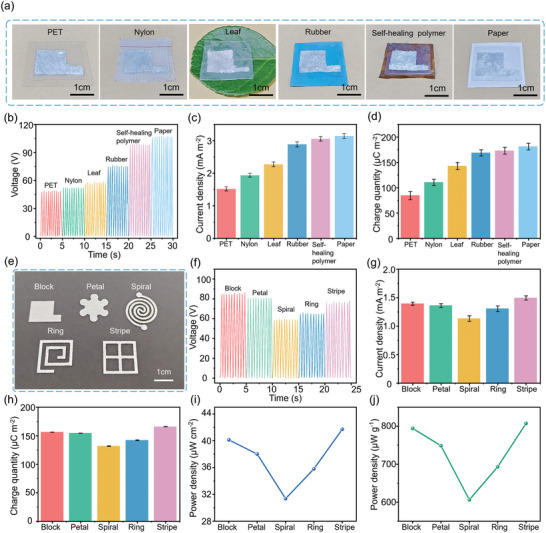
Performance characterization of the bGaIn‐based TENG. a) Optical images and b) output voltage, c) current, and d) charge density of the bGaIn‐based TENG on different substrates. e) Optical images and f) output voltage, g) current, h) charge density, and power density over i) area and j) weight of the bGaIn‐based TENG electrodes with different shapes (tested at 2 Hz and 5 N).

Despite the excellent performance of the stretchable TENG with LM filled in the microfluidic channels, the device is associated with a large footprint/weight from the microfluidics. It is also difficult to integrate the device on different substrates due to the high surface tension and low substrate adhesion of LMs. As an alternative solution, the solid‐liquid dual‐phase bGaIn from the oxidization of liquid EGaIn reduces surface tension and enhances adhesion, allowing facile (stencil) printing on diverse substrates (e.g., fabric, plastic, plant, rubber, self‐healing polymers, paper) (**Figure** **5**a; Table S2, Supporting Information).

The comparison in the output voltage of the TENGs on diverse substrates for a loading force of 5 N at 2 Hz (Figure 5b–d) reveals the difference in the surface adhesion, which can affect the contact area and formed gaps between the electrodes and the friction layer at the interface. With the same external force, the substrate with a stronger adhesion gives a larger contact area and a greater frictional resistance during the contact process to produce a larger electrical output.^[^ ^69^, ^70^ ^]^ In addition, the small gap between bGaIn and the substrate during the printing process may also have influence on the electrical output characteristics,^[^ ^71^ ^]^ which deserves to be discussed in the future study.

The bGaIn‐based TENGs on the paper substrate also show a maximum output voltage of 101.7 V, current density of 3.1 mA m^−2^, and transferred charge density of 181.4µC m^−2^ at 2 Hz in the range from 1 to 5 Hz for a loading force of 5 N (Figure S17a, Supporting Information). The increase in the loading force (at 2 Hz) also results in enhanced electrical output characteristics to achieve the peak voltage of 179.2 V, current density of 5.7 mA m^−2^, and transferred charge density of 233.1 µC m^−2^ at 13 N (Figure S17b, Supporting Information). The bGaIn‐based TENG exhibits the maximum peak power density of 23.6 µW cm^−2^ at an external load resistance of 50 MΩ (Figure S18a,b, Supporting Information) and can charge different capacitors from 1 to 10 µF (Figure S18c, Supporting Information), which have benefits compared with similar work (Table S3, Supporting Information). The bGaIn‐based TENG with stable performance over 10000 cycles (Figure S19, Supporting Information).

The comparison between bGaIn electrodes with different shapes (i.e., block, petal, spiral, ring, and stripe) on paper (Figure 5e) reveals the means to create stretchable bGaIn‐based TENGs with enhanced performance parameters per unit area and weight. While TENG with the block shape shows the largest voltage output of 84.4 V, the one with the stripe electrode exhibits the largest current density and charge density (Figure 5f–h), likely resulting from the varying interfacial contact.^[^ ^72^ ^]^ Additionally, the smaller size and amount of bGaIn in the stripe electrode result in enhanced power output normalized by both the size and weight (Figure 5i,j; Table S4, Supporting Information). Considering the enhanced stretchability of TENG with spiral and ring electrodes (Figure S20, Supporting Information), the electrode shapes could be further optimized for simultaneously achieving high mechanical and electrical properties.

### 2.6 Applications of the TENG Based on Liquid Metal

**Figure 6:**
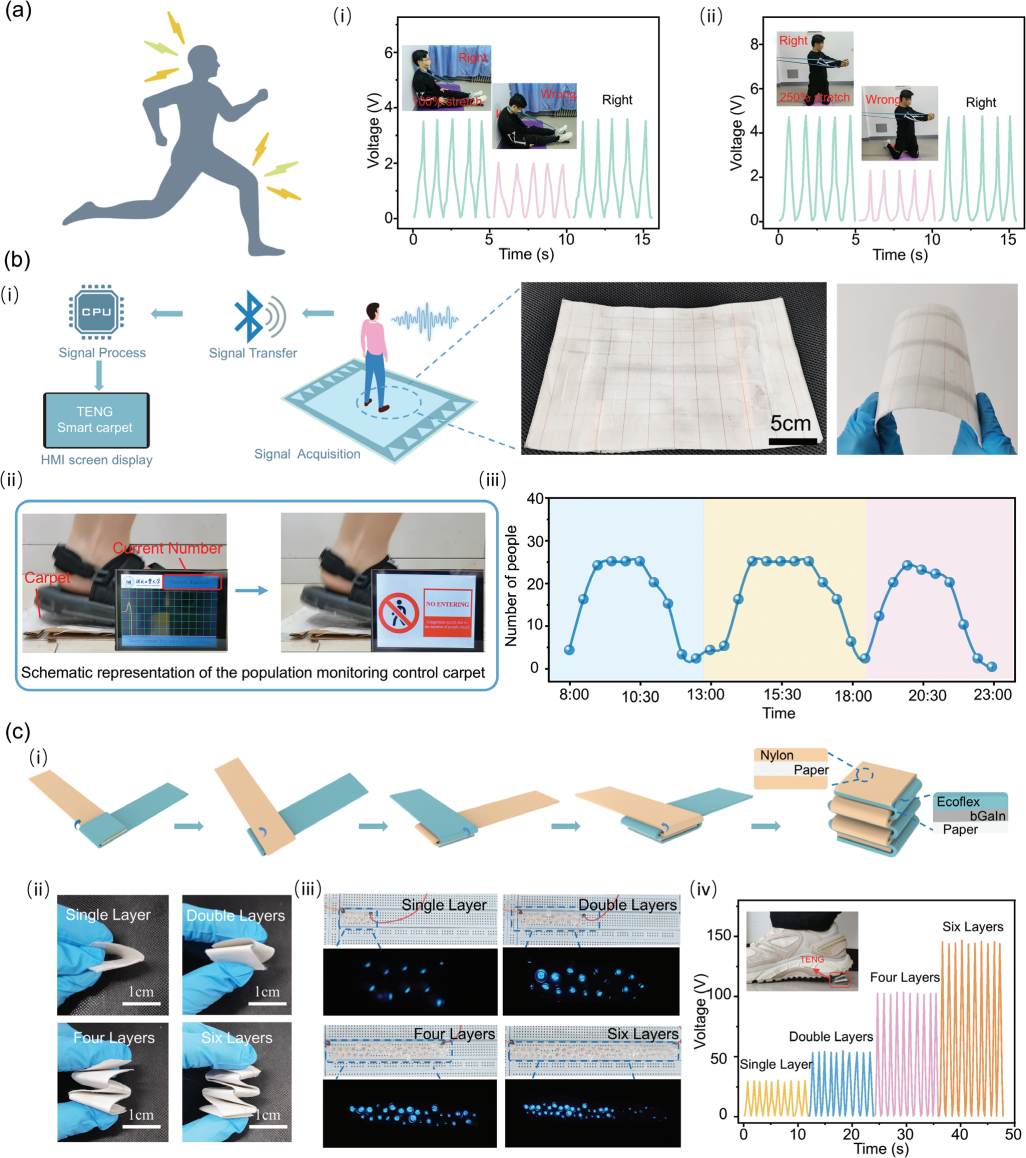
The application of the TENG based on liquid metal with varying phases. a) Monitoring of physical exercise and human motion with the EGaIn‐based TENG. i) Anti‐flexion exercises. ii) Kneeling rotation exercise. b) Smart carpet system for monitoring people stream based on bGaIn printing on nylon fabrics. i) Schematic diagram. ii) Physical demonstration of crowd control. iii) Monitoring of laboratory occupancy. c) Paper‐based foldable TENG based on bGaIn. i) Folding Processes. ii) Optical image of the bGaIn‐based foldable TENG (with 1, 2, 4, and 6 layers). iii) Lights up the LEDs. iv) Monitors foot information.

The high stretchability of EGaIn‐based TENG allows integration on elastic bands to monitor physical exercise for maintaining a healthy lifestyle (Figure S21, Supporting Information). For instance, the smart elastic band could ensure the vertical position of the upper back in anti‐flexion exercises as correct postures give an output voltage of 3.8 V with the local strain reaching 100% versus 1.9 V in the wrong posture for the 24‐year‐old subject (height 173 cm, weight 70 kg) (**Figure** **6**a–i). During the kneeling rotation exercise with lumber rotation and the local stretching of 250%, the elastic band gives a voltage output of 4.5 V (or 2.3 V) in the correct (or incorrect) posture (Figure 6a‐ii). This was attributed to the change in the stretch rate of the TENG under different training motions. Placing the stretchable EGaIn‐based TENG on different body parts also simply monitors the movement of various body parts, including finger flexion angle (Figure S22a, Supporting Information) and elbow and knee flexion angle during exercise (Figure S22b,c, Supporting Information) by the electrical output characteristics resulting from the difference contact area between the TENGs and skin. With the TENG under the shoe, running with high frequency can be differentiated from jogging with medium frequency and walking with lower frequency (Figure S22d, Supporting Information).

The bGaIn‐based TENG on diverse substrates further opens up application opportunities. Integration of the striped bGaIn‐based TENG on the back of the carpet using nylon fabric as a positive friction layer with signal acquisition, wireless transmission, data processing, and display components (Figure S23a,b, Supporting Information) results in a wireless self‐powered intelligent carpet (Figure 6b‐i). With the weight of the human subjects ranging from 40 to 80 kg, the people stream or the number of people passing on the carpet is accurately monitored (Figure S23c, Supporting Information). Besides detecting the number of people who stepped on the carpet with the increased electrical signal (Figure 6b‐ii; Movie S5, Supporting Information), the intelligent carpet can also be used in laboratories or classrooms for activity monitoring (Figure 6b‐iii).

The bGaIn‐based TENGs on plant leaves can monitor wind speed and rainfall in the natural environment (Figure S24, Supporting Information), without nucleation or condensation of water molecules due to the hydrophobic Ecoflex. In addition, the bGaIn‐based TENGs on the nitrile gloves (at each finger joint) provide the opportunity for gesture recognition such as the “like” or “yes” gestures (Figure S25, Supporting Information).

The TENG on paper combined with origami folding (Figure 6c‐i) can stack multi‐layers (1, 2, 4, or 6 layers) (Figure 6c‐ii) to power a large number (10, 24, 42 or 60) of LEDs (Figure 6c‐iii; Movie S6, Supporting Information). When integrated on the shoe sole area, the origami‐folded TENG at a small device footprint can also provide increased mechanical strength and support for analyzing plantar information (Figure 6c‐iv, body weight of 70 kg). With a voltage regulator circuit (Figure S26a, Supporting Information) to avoid distortion of the manual drive waveform (from varied frequency of 2–3 Hz and the load force of 5–10 N), the electrical output voltages can be accurately measured to be 0.75, 1.72, 2.49, and 3.75 V for the TENG with 1, 2, 4, and 6 layers (Figure S26b, Supporting Information).

**Figure 7:**
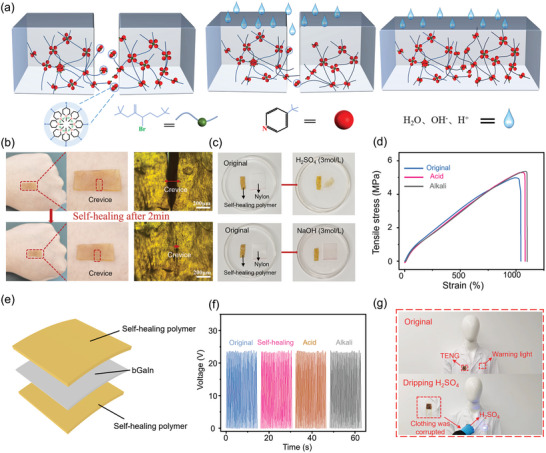
Performance characterization of the bGaIn‐based TENG in extreme environments. a) Mechanisms of self‐healing and acidic/alkaline resistance. Experimental verification of b) self‐healing and c) acidic/alkaline resistance. d) Mechanical properties after immersion in acid and alkali. e) Schematic diagram of acidic‐ and alkaline‐resistant TENG. f) Electrical performance of TENG in extreme environments. g) Alarm device for use in acidic and alkaline environments.

The acidic/alkaline resistance and healing mechanism of acidic‐/alkaline‐resistant polymers are provided by grafting tert‐butyl pyridine (TB) on the chain of bromobutyl rubber (BIIR).^[^ ^73^ ^]^ The hydrophobic TB tends to organize on the surface of ionic aggregates to form protective layers, repelling water molecules and H^+^/OH^−^ ions (**Figure** **7**a). In addition, the ionic bonds formed between TB and allyl bromide could be dynamically exchanged and reconfigured in the physical cross‐linking structure, allowing the material to self heal under molecular motion. The relatively small changes in the stress‐strain curve after multiple cutting‐healing cycles indicate stable performance over repeated healing (Figure S27, Supporting Information). As a result, the self‐healing acidic‐ and alkaline‐resistant polymers can rapidly (within 2 min) close the gap after cutting (Figure 7b), allowing for rapid self‐healing when combined with the printed bGaIn circuitry (Figure S28, Supporting Information). Stable surface morphology and mechanical properties are evident after immersing in H_2_SO_4_ and NaOH (3 mol L^−1^) solutions for 10 min and 24 h (Figure 7c,d and Movie 7, Supporting Information). The electrical properties of TENG with the bGaIn sandwiched between two self‐healing acidic‐/alkaline‐resistant polymers (Figure 7e) also remain stable after cutting/healing and immersing in acidic‐ and alkaline‐solutions, demonstrating the use in extreme environments (Figure 7f). The long‐term stability of the device is confirmed by immersing it in acidic and alkaline solutions for 24 hours (Figure S29, Supporting Information) and applying 10000 load cycles (Figure S30, Supporting Information). Attaching the designed TENG to the rescue suit can sense drops of acidic/alkaline solutions or manual tapping to send an alarm and notify the rescue team for support (Figure 7g; Figure S31 and Movie S8, Supporting Information).

## 3 Conclusion

In conclusion, this work presents the design and demonstration of stretchable and substrate‐adaptable TENGs based on liquid metal with different crystalline phases. The EGaIn‐based TENG with a stretchability of up to 867% integrated on the elastic band can be used to monitor physical exercise or large human movements. The bGaIn‐based TENG on diverse substrates with different electrode shapes further reduces the device footprint and weight to go beyond gesture recognition and windspeed/rainfall monitoring to smart carpet for population stream monitoring, foldable TENGs in space‐limited applications, and acidic/alkaline‐resistant rescue monitors. The results from this work provide promising demonstrations for the application of TENG based on the liquid metal with varying phases, including health monitoring, self‐powered sensing in extreme environments, and human‐robot collaboration.

## 4 Experimental Section

### Fabrication of Flexible, Stretchable TENG

The molds with different structural parameters designed by 3D modeling software (Solidworks) were first printed by a 3D printing machine (Raise3D Pro2). Next, parts A (base polymer) and B (curing agent) of the silicone rubber solution (EcoflexTM00‐50, Smooth‐on, Inc.) were magnetically stirred for 1 min at a speed of 1500 rpm at a ratio of 1:1 by volume. Injecting 1.5 mL of the well‐mixed solution into the molds for the serpentine textured base and the planar textured surface was followed by placing molds in a vacuum drying oven for 2 min to remove air bubbles and curing at 60 °C. After peeling the cured silicone rubber from the molds and placing the copper wires in the serpentine structure, trace amounts of uncured silicone rubber were used to bond the two and form the microfluidic structure. Finally, 0.2 mL of liquid metal EGaIn (75.5% Ga, 24.5% In, Dongguan Wochang Metal Products Co., Ltd.) was injected into the microfluidic structure with a syringe from one end, with another syringe at the other end for pumping. The liquid metal bonded with the embedded wires yielded a flexible stretchable TENG.

### Production of bGaIn Liquid Metal Materials

After mixing EGaIn with anhydrous ethanol (99.99 percent purity) at a ratio of 90 mg:1 mL, placement in an ultrasonic cleaner for 10 min broke down the large droplets into smaller particles to form a grey suspension. Processing the suspension in a 720 W ultrasonic cell crusher for 45 min ensured well mixing. Next, the well‐mixed solution was placed in a spray gun for even spraying onto the Si wafer, followed by drying in a drying oven at 85 °C for 60 s. Rapid evaporation of the anhydrous ethanol at high temperatures and surface oxidation of the fine EGaIn particles to Ga_2_O_3_ changed color from dark to light. Solid Ga_2_O_3_ encapsulated the liquid EGaIn to form a solid‐liquid biphasic film, with the thickness increasing with repeated spraying (15 times), which could be easily scraped off the Si surface with a scraper.

### Preparation of Ecoflex Thin Films

Solutions A and B of the Ecoflex were mixed at a volume ratio of 1:1 and stirred with a magnetic stirrer at a speed of 1500 rpm for 1 min. The mixed solution was then poured onto a PET substrate and coated with a film applicator for a thickness of 500 µm. The evenly coated Ecoflex was placed in a vacuum‐drying oven for 2 min to remove air bubbles and then heated at 60 °C for 2 h for curing, followed by peeling off from the PET substrate.

### Preparation of Lightweight TENG on Diverse Substrates

Laser cutting of the PET substrate first created the template with diverse shapes, such as a square of 2 cm × 2 cm. After placing the template over a variety of substrates, applying bGaIn over the entire surface using a spatula allowed the pattern formation through the template opening. Removing the template and then encapsulating it with the Ecoflex silicone rubber resulted in the TENG on the target substrate.

### Characterizations and Measurements

The SEM images were obtained using a field emission SEM (TESCAN, GAIA3). The XRD spectra were measured by a D8 Advance (Bruker) X‐ray diffractometer. The 180° peel test and uniaxial tensile strain were measured with a multi‐substrate adaptive testing machine (CTM‐1, Jinan Liangong Testing Technology Co., Ltd., China). The flexible electronic tester (FT2000) was used to apply tension to the device and the excitation system (Donghua Testing Technology Co., Ltd., China) was used to provide simulated vibration at given frequencies and load force. The electrostatic meter (Keithley 6514, USA) was used to measure open circuit voltages and transferred charges, whereas an electrochemical workstation (Vertex.C.EIS, Ivium Technologies BV, The Netherlands) was used to measure short‐circuit current. A multichannel oscilloscope (MDO 5104B, Tektronix, USA with the probes used as working and reference probes) with a 10 MΩ probe was used to capture the voltage for gesture recognition. Using Origin software to create data graphs.

## Conflict of Interest

The authors declare no conflict of interest.

## Author Contributions

L.Y. and H.C. initiated the project. L.Y. supervised the studies. L.G. and Z.W. led the experiments and collected the overall data. L.G., Z.W., and X.C. contributed to the device design, fabrication, and characterization. L.G. and X.J. contributed to the application exploration of the device. L.G., Z.W., L.Y, and H.C contributed to data analysis and cowrote the paper. All authors provided feedback on the manuscript.

## Supporting information

Supporting Information

Supplemental Video 1

Supplemental Video 2

Supplemental Video 3

Supplemental Video 4

Supplemental Video 5

Supplemental Video 6

Supplemental Video 7

Supplemental Video 8

## Data Availability

The data that support the findings of this study are available from the corresponding author upon reasonable request.
