# Supplementary material for: Stretchable Triboelectric Nanogenerator Based on Liquid Metal with Varying Phases
Source: Adv Sci (Weinh). 2024 Aug 13;11(39):2405792. doi: 10.1002/advs.202405792 (PMC11497018; doi:10.1002/advs.202405792)
Supplement: Supplementary file 1 — Supporting Information [file ADVS-11-2405792-s001.docx]

Supporting Information

Stretchable Triboelectric Nanogenerator Based on Liquid Metal with Varying Phases

Li Yang^1,^ *, Langang Guo^2^, Zihan Wang^2^, Chuizhou Meng^2^, Jinrong Wu^5^, Xue Chen^3^, Abdullah Abu Musa^4^, Xiaoqi Jiang^1^, Huanyu Cheng^4,^ *.

^1^ State Key Laboratory of Reliability and Intelligence of Electrical Equipment, School of Health Sciences and Biomedical Engineering, Hebei University of Technology, Tianjin 300130, China.

^2^ State Key Laboratory for Reliability and Intelligence of Electrical Equipment, Hebei Key Laboratory of Smart Sensing and Human-Robot Interaction, School of Mechanical Engineering, Hebei University of Technology, Tianjin 300401, China.

^3^ State Key Laboratory of Reliability and Intelligence of Electrical Equipment, Key Laboratory of Bioelectromagnetics and Neuroengineering of Hebei Province, School of Electrical Engineering, Hebei University of Technology, Tianjin 300130, China.

^4^ Department of Engineering Science and Mechanics, The Pennsylvania State University, University Park, 16802, USA.

^5^ State Key Laboratory of Polymer Material Engineering, College of Polymer Science and Engineering, Sichuan University, Chengdu 610065, China.

**Corresponding Author**

*E-mail: yangli5781@126.com.

*E-mail: huanyu.cheng@psu.edu.


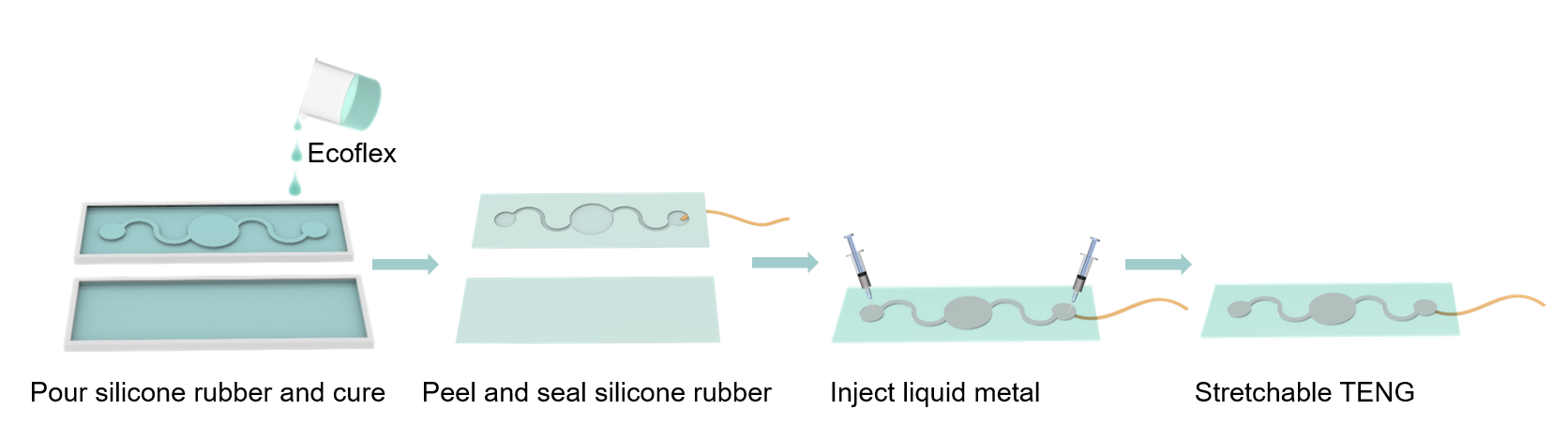


**Figure S1.** Schematic showing the fabrication process of the stretchable EGaIn-based TENG.


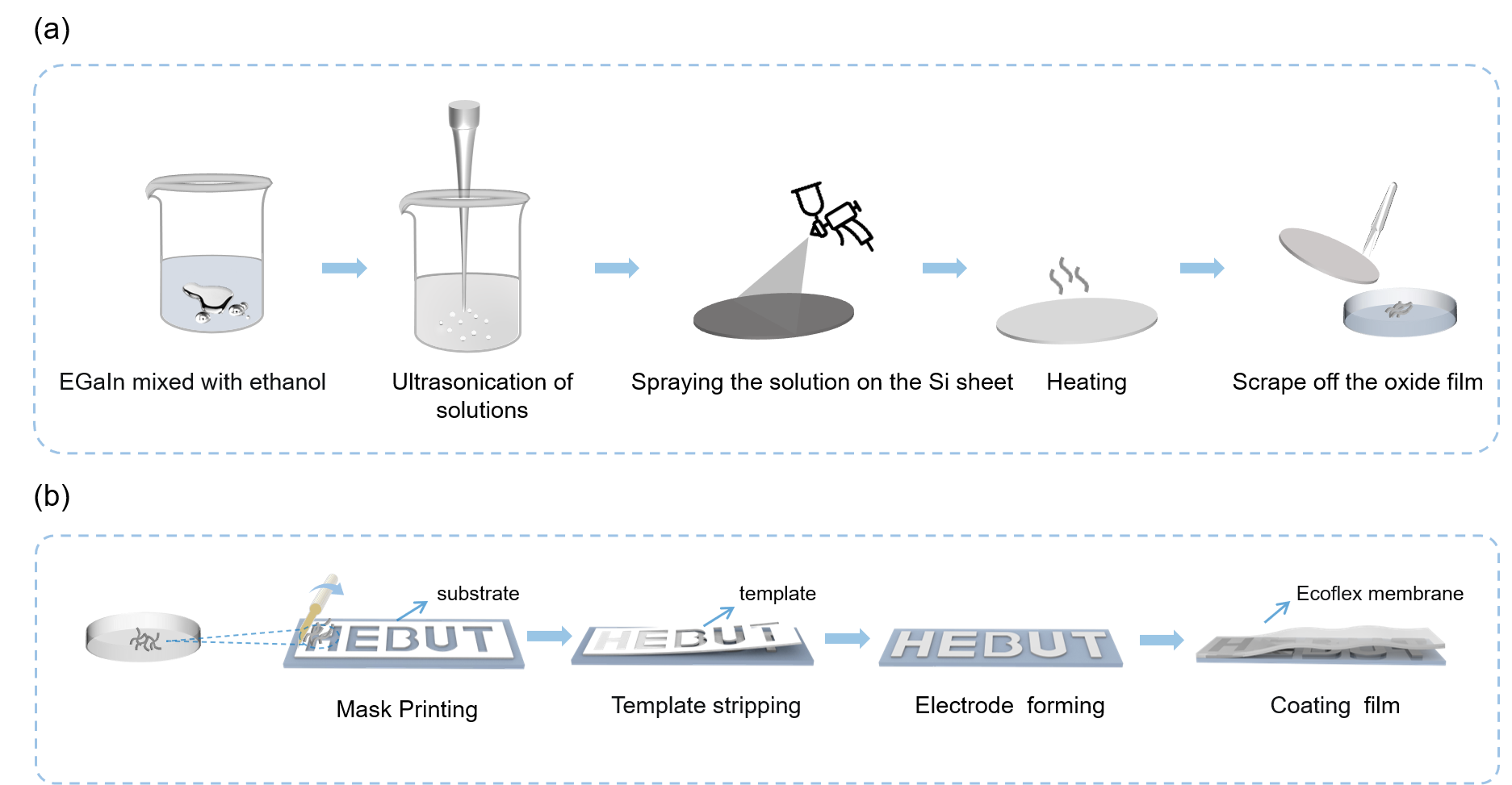


**Figure S2.** a) Oxidation of EGaIn to prepare bGaIn and b) its printing on diverse substrates to yield bGaIn-based TENG.


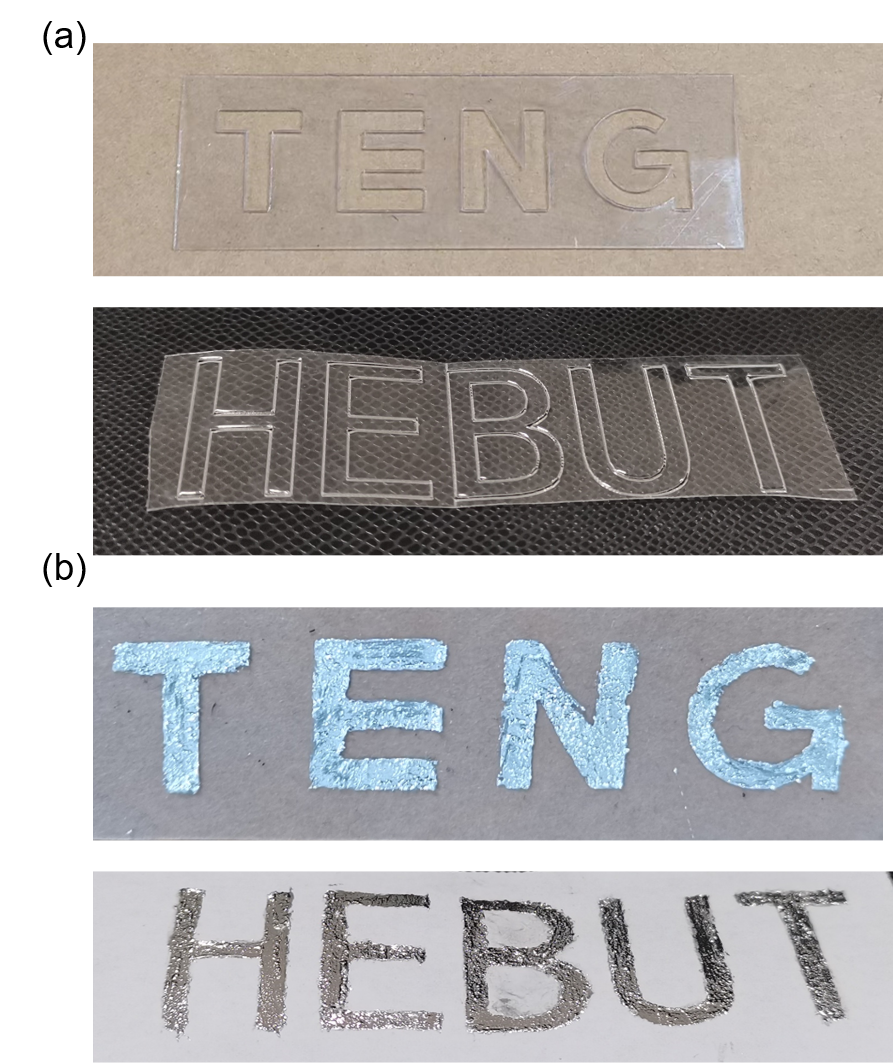


**Figure S3.** Optical images of a) masks and b) printed patterns on silicone rubber (top) and paper(bottom) substrates.


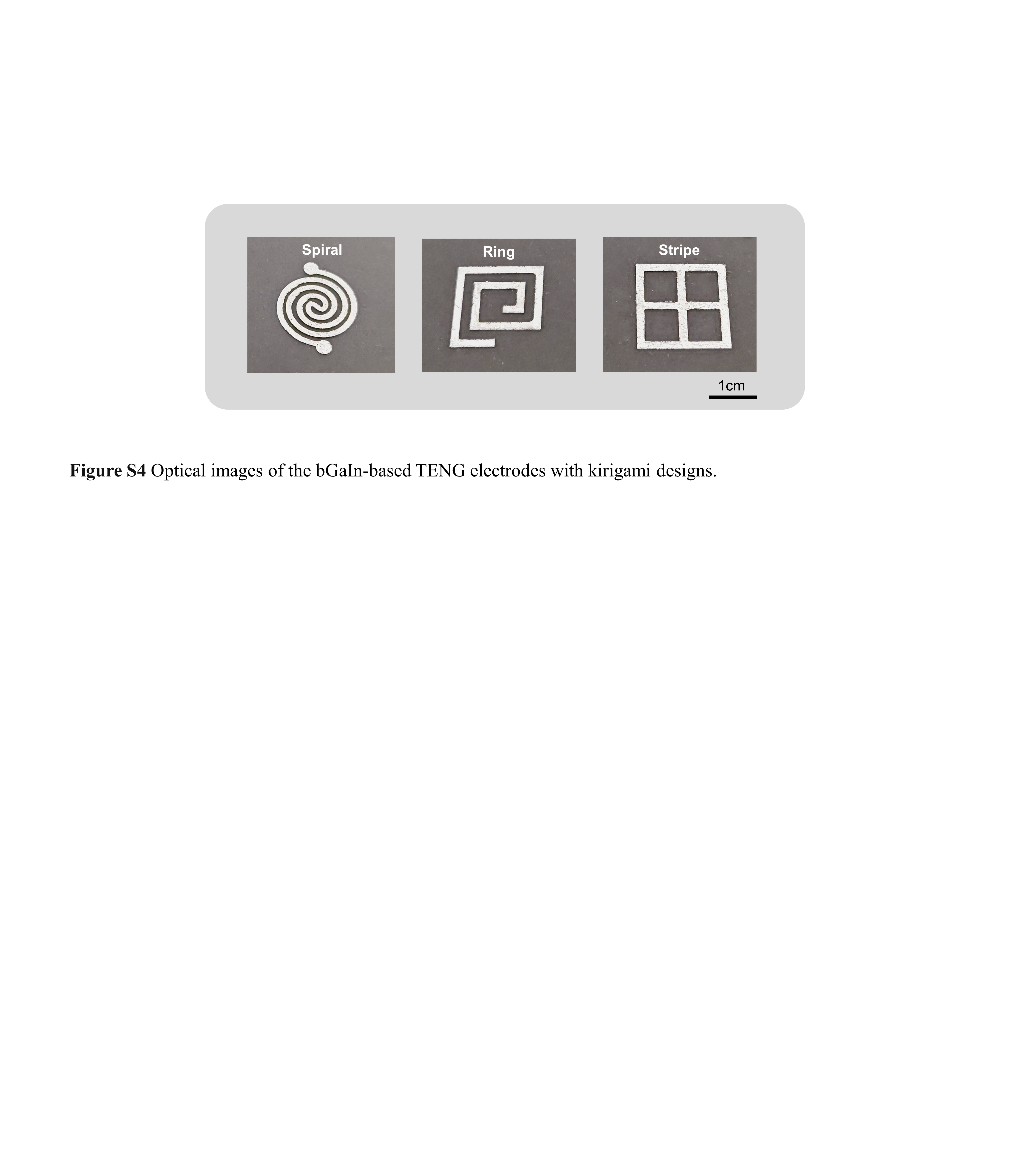


**Figure S4.** Optical images of the bGaIn-based TENG electrodes with kirigami designs (i.e., spiral, ring, and stripe).


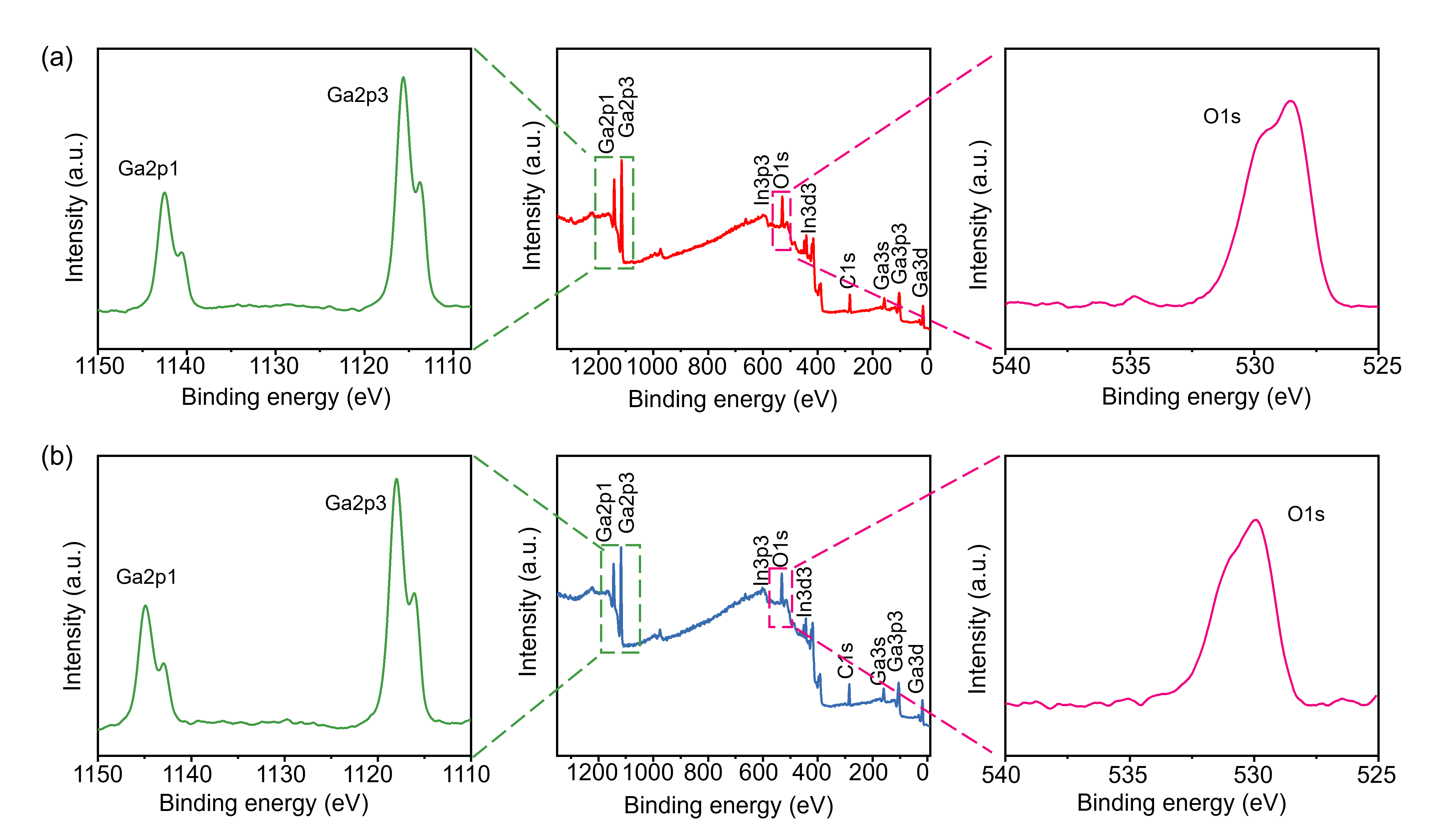


**Figure S5.** The XPS characterization of a) EGaIn and b) bGaIn.


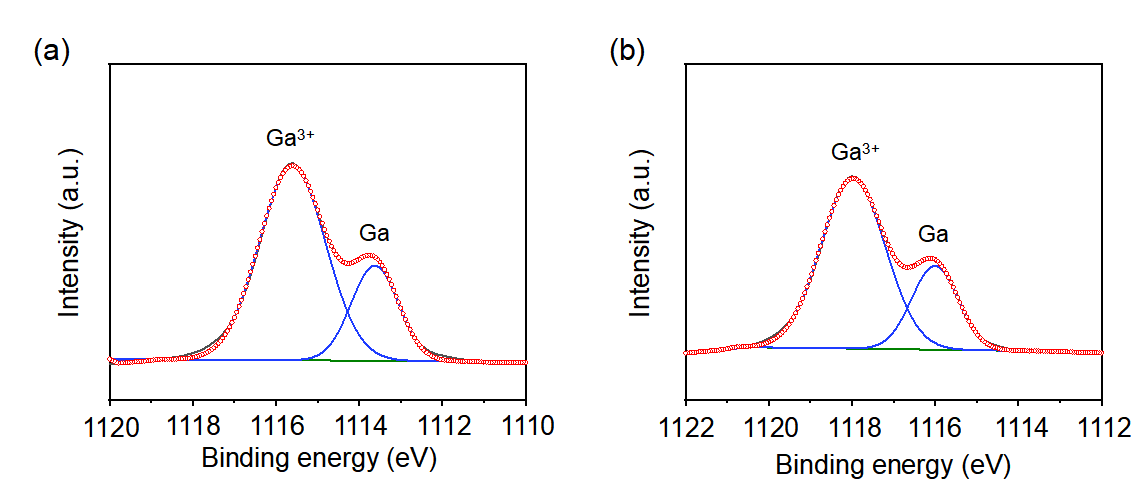


**Figure S6**. The XPS analysis of a) EGaIn and b) bGaIn with Ga^3+^ and Ga peaks identified.


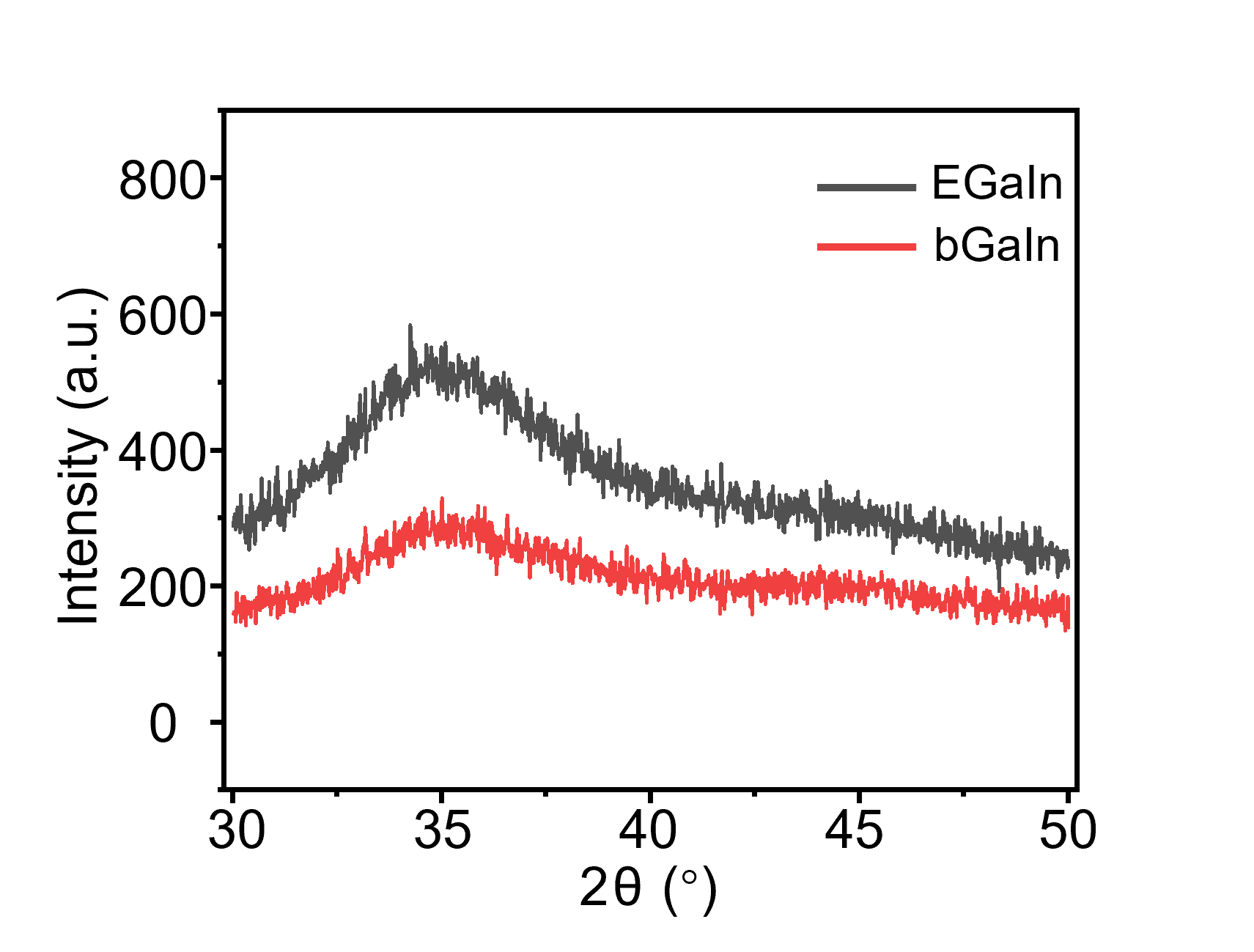


**Figure S7**. The XRD characterization test of EGaIn and bGaIn.


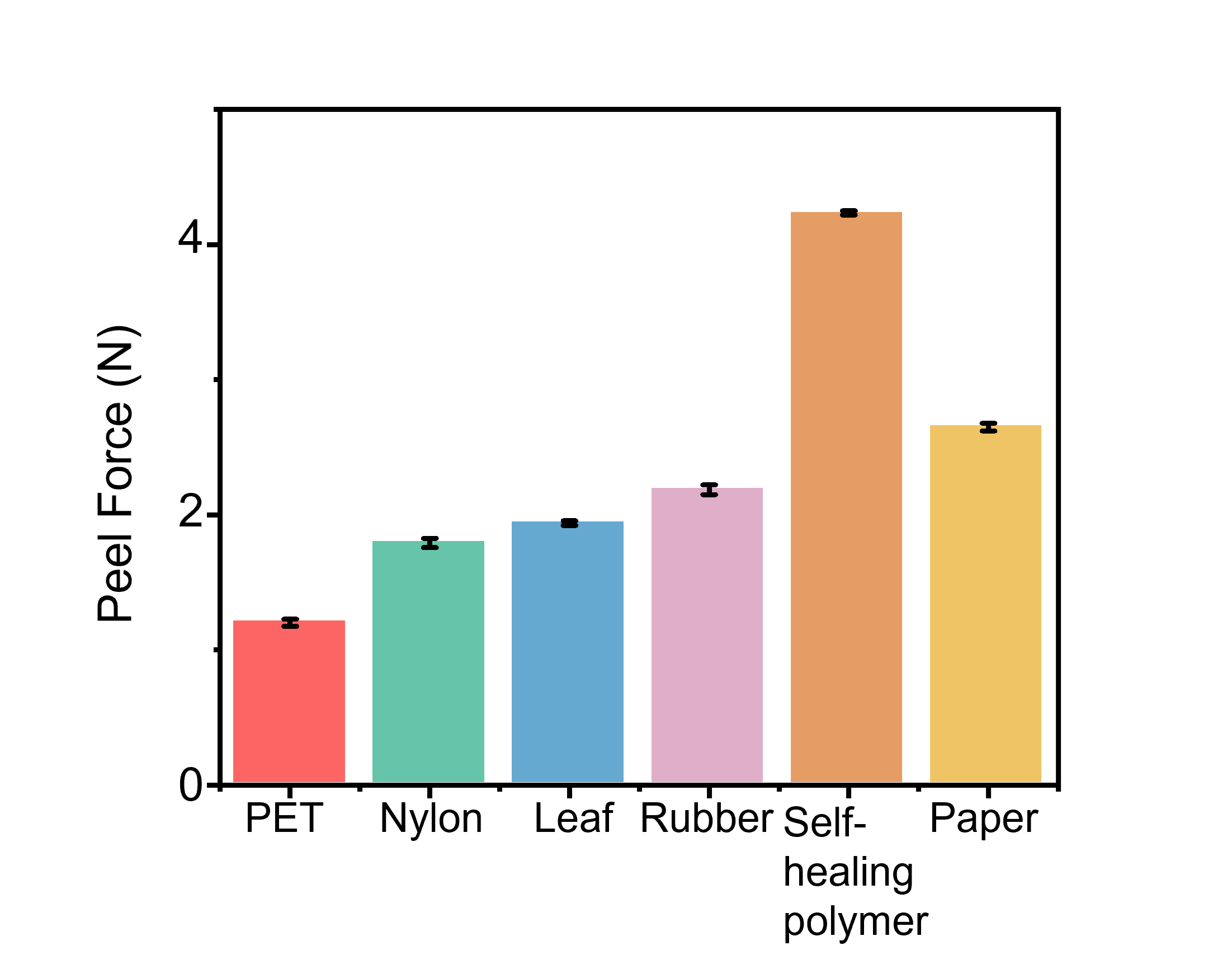


**Figure S8.** The adhesion test under the same conditions on different substrates.


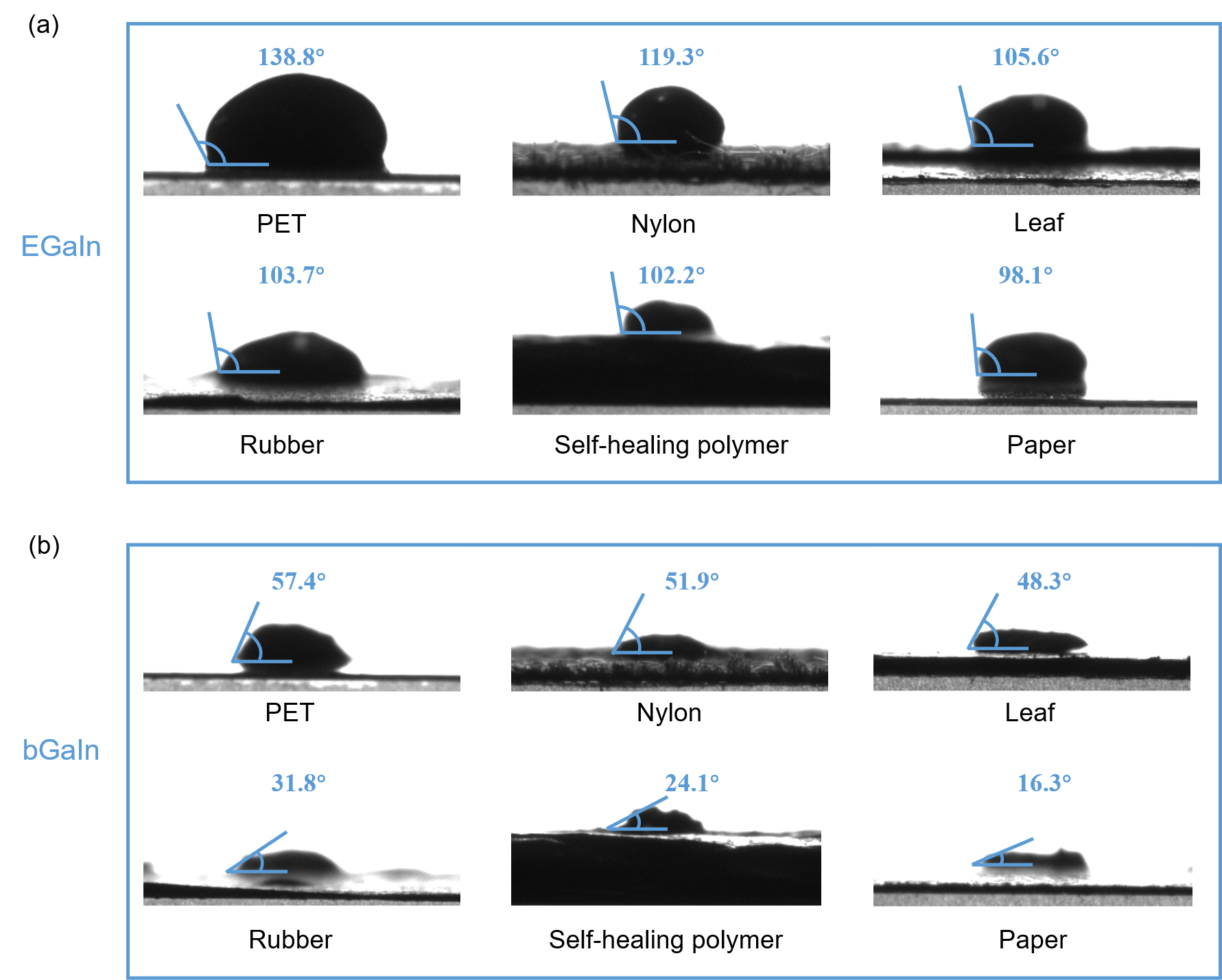


**Figure S9.** Contact angles of a) EGaIn and b) bGaIn on different substrates: PET, nylon, leaf, rubber, self-healing polyer, and paper.


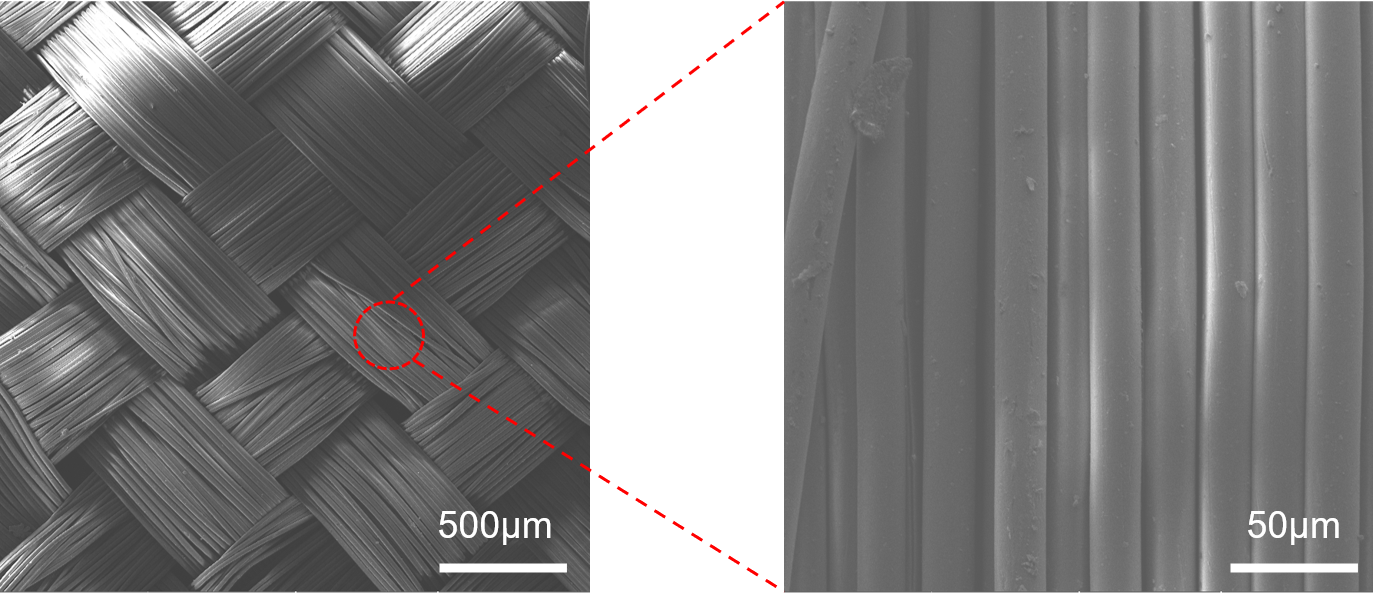


**Figure S10.** SEM images of the nylon fabric surface.


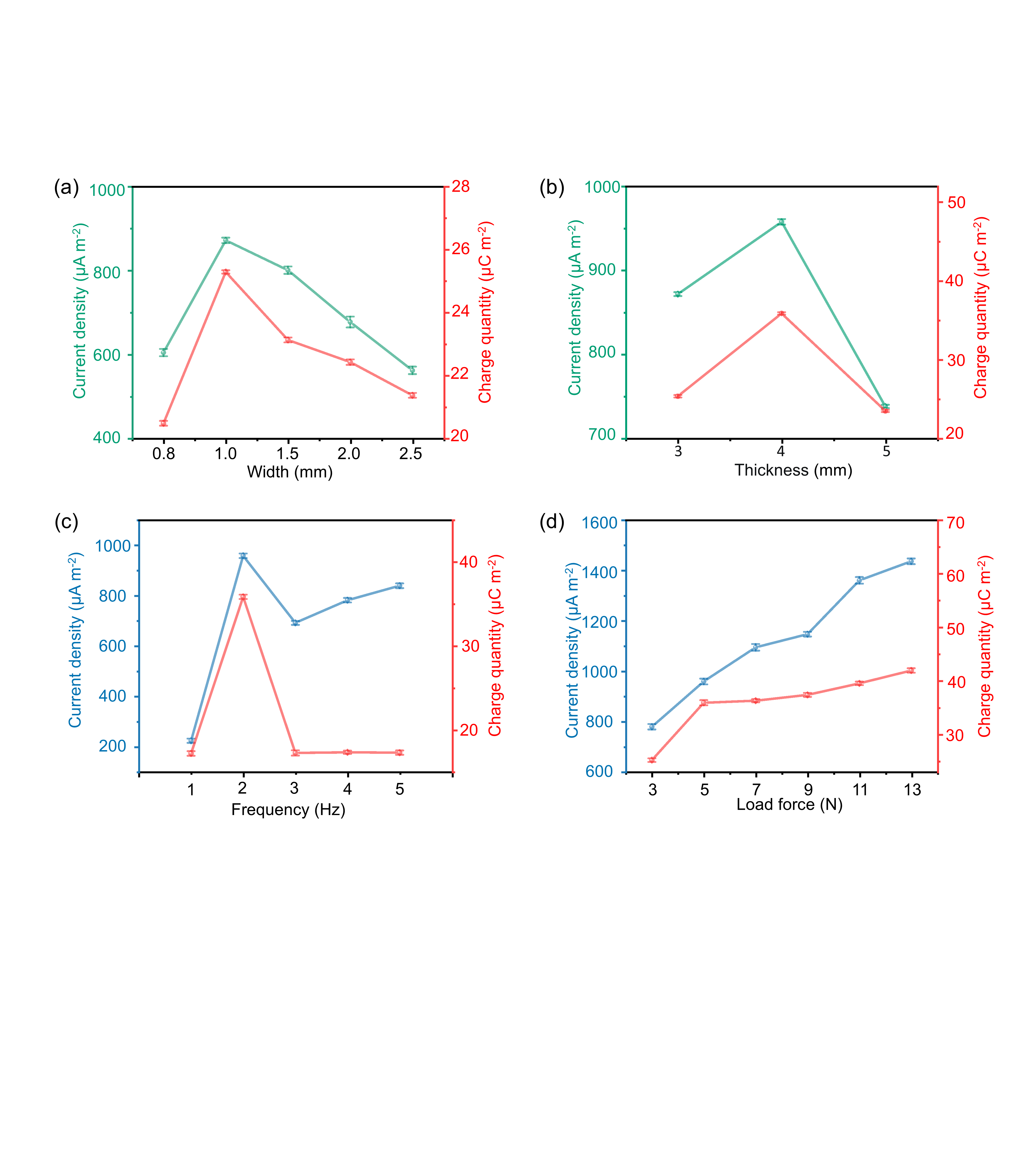


**Figure S11.** Comparison in the short-circuit current and transfer charge density of stretchable TENG with different a) widths, b) thicknesses, c) frequencies, and d) loading forces.


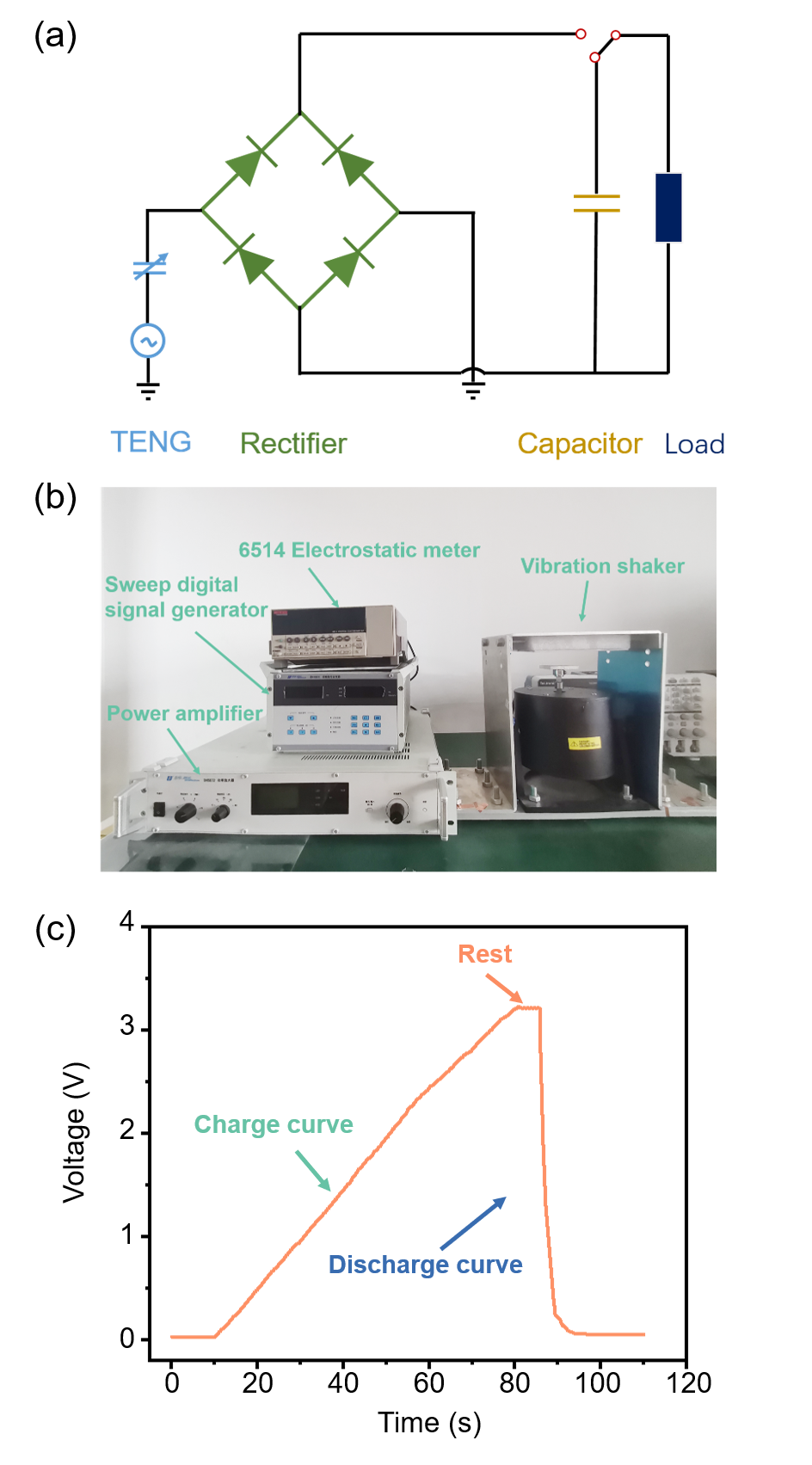


**Figure S12.** a) Power management circuit for energy harvesting from the TENG. b) Optical image of the experimental setup to apply load and measure TENG ouputs. c) Charging curve of the commercial capacitor of 4.7 uF.


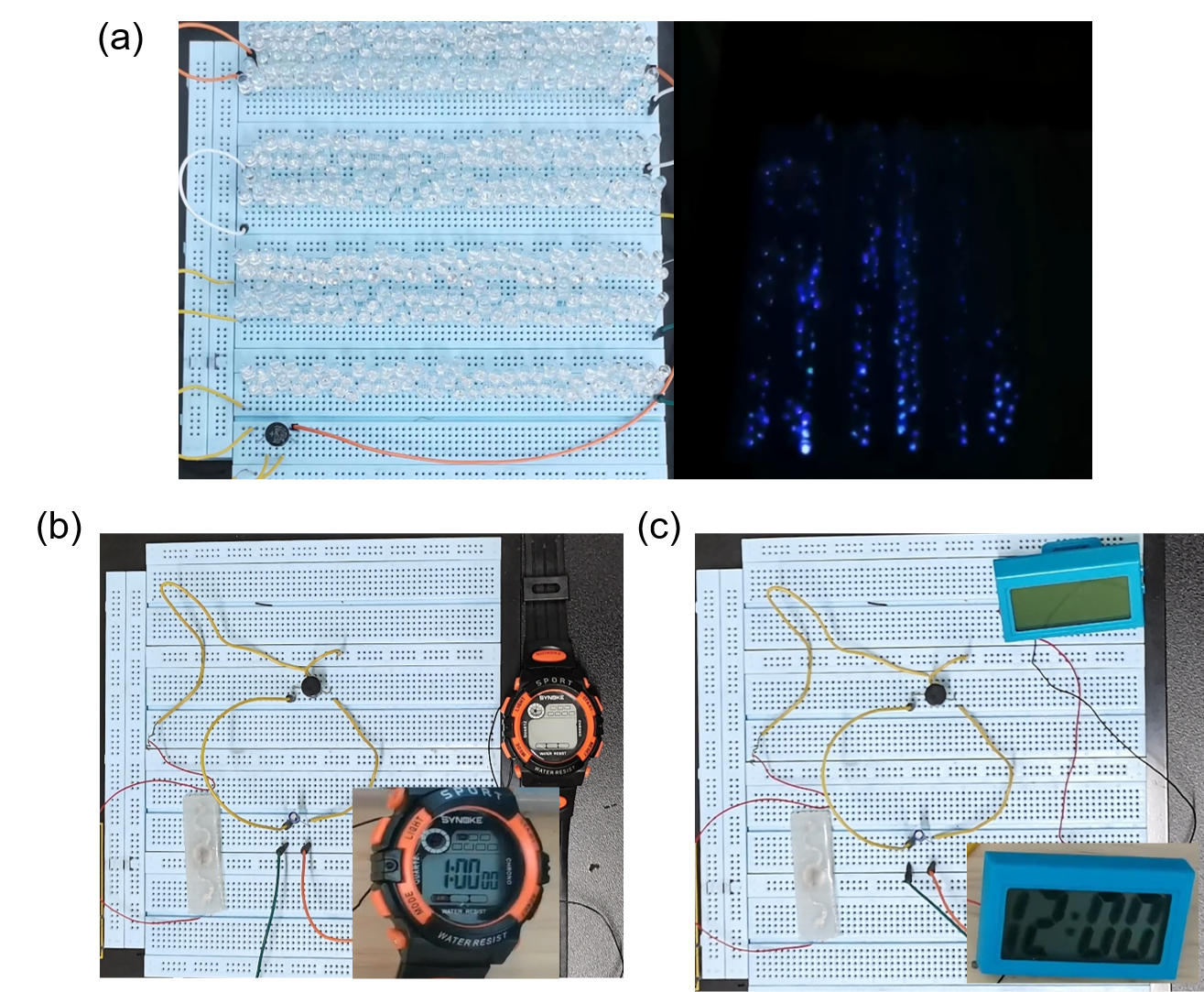


**Figure S13.** Demonstration of the harvested energy from the TENG to drive a) 430 LEDs, b) a low-power electronic clock, and c) an electronic watch.


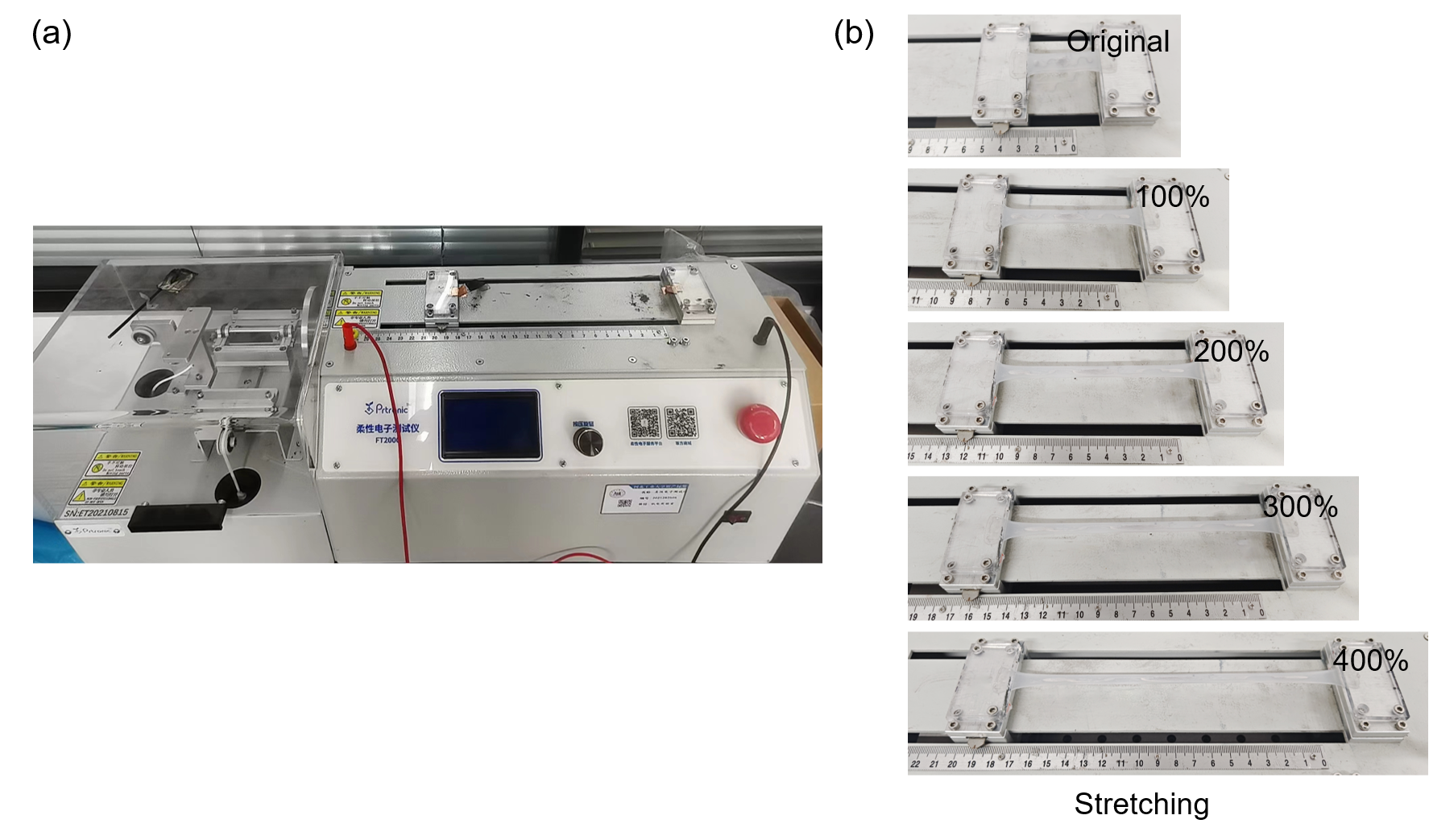


**Figure S14.** a) A flexible electronic tester to apply cyclic tensile strains and b) the device upon stretching of 400%.


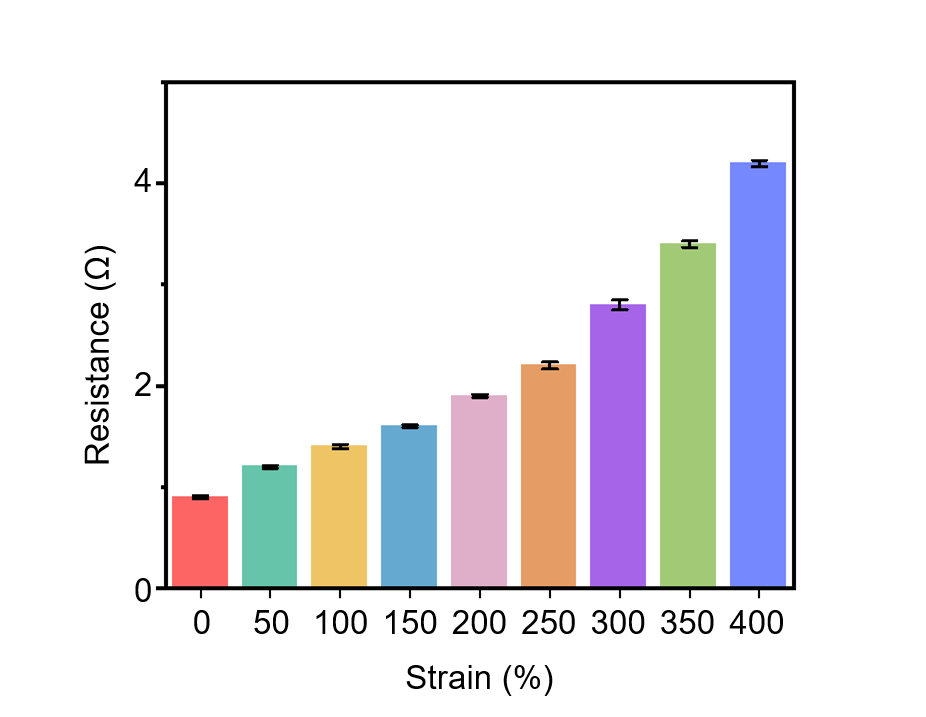


**Figure S15.** Resistance changes of EGaIn upon stretching to 400%.


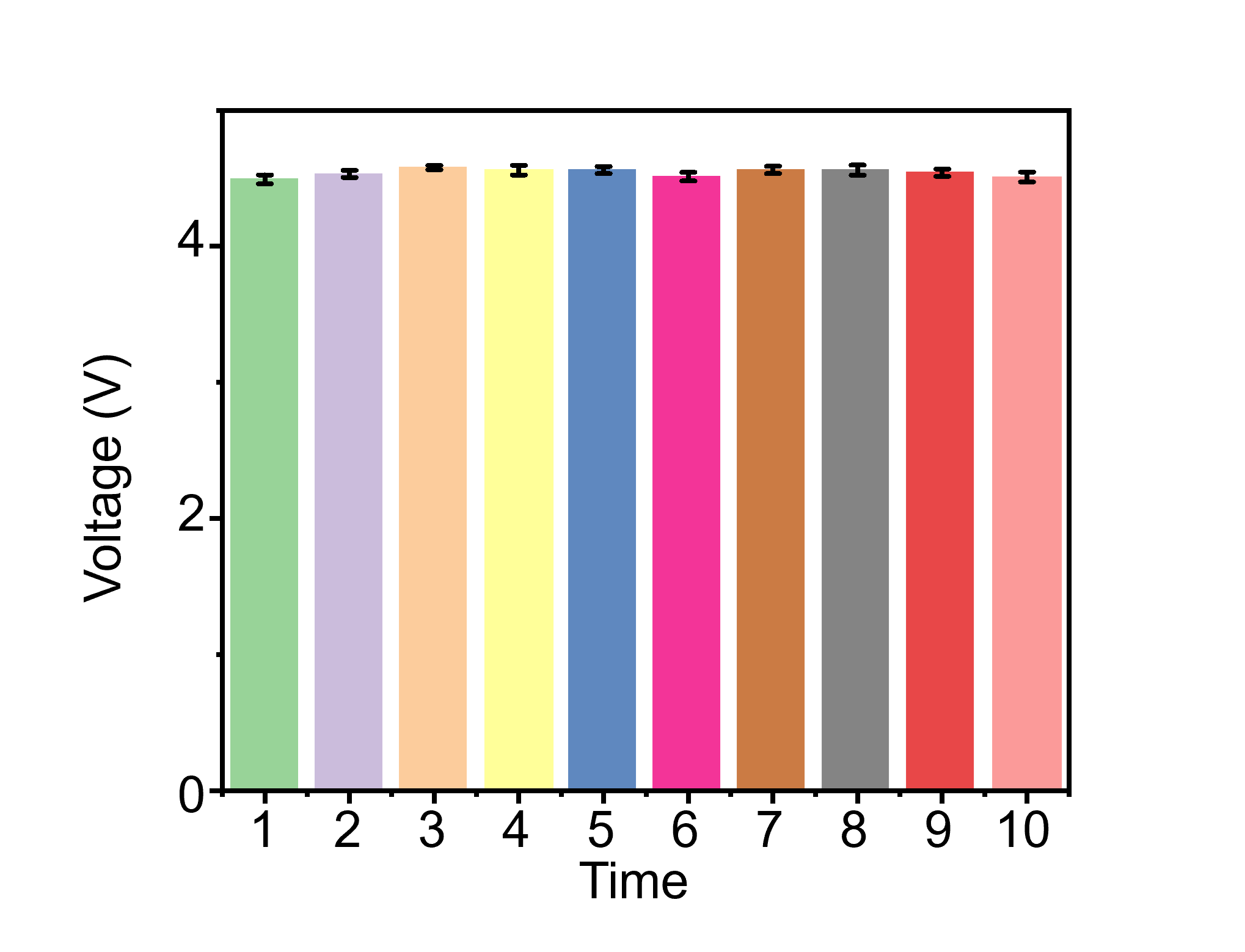


**Figure S16**. The voltage signal under 10 measurements at the tensile strength of 250%.


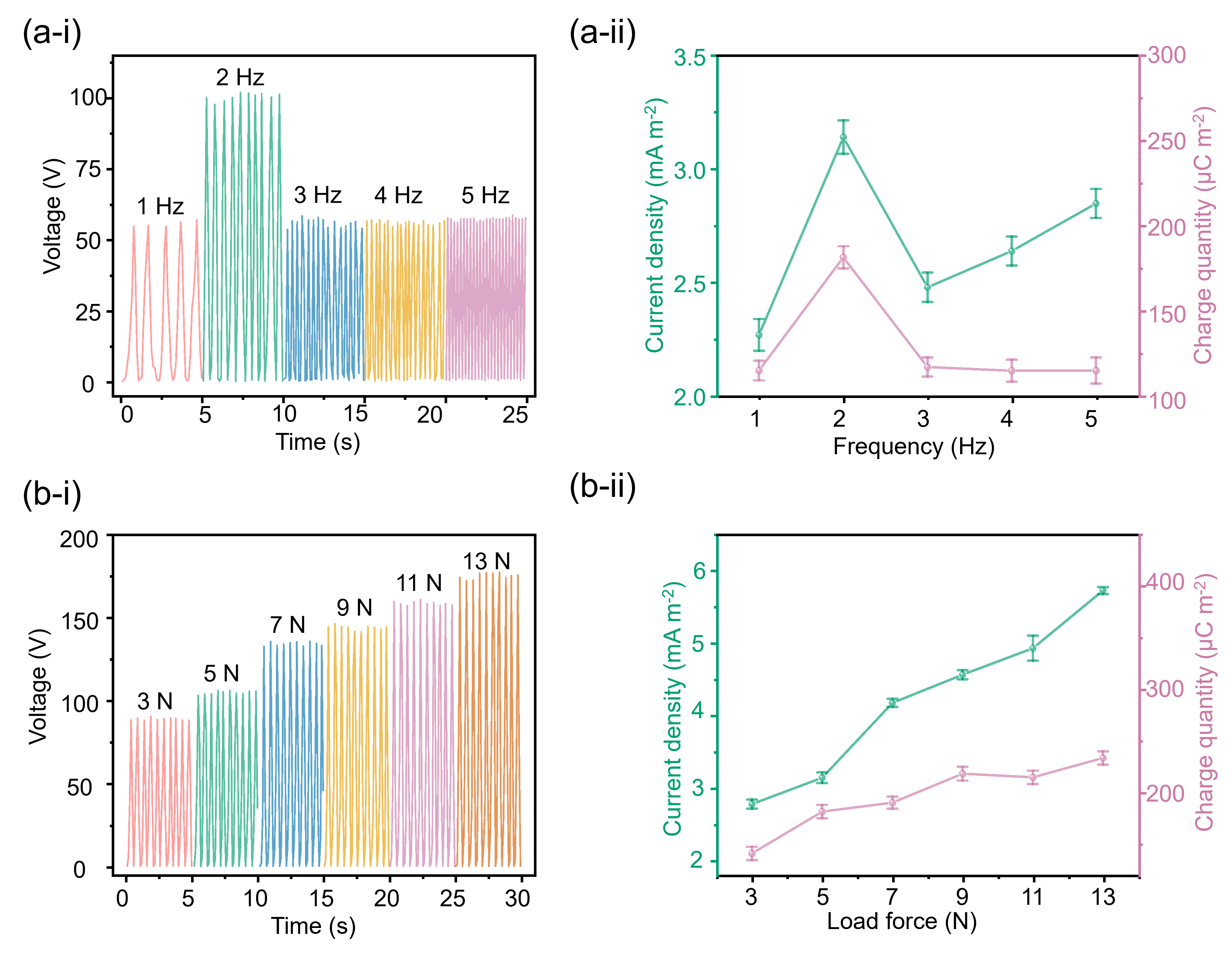


**Figure S17.** The effect of loading a) frequency and b) force on the i) voltage, ii) current and charge density of the bGaIn-based TENG on paper.


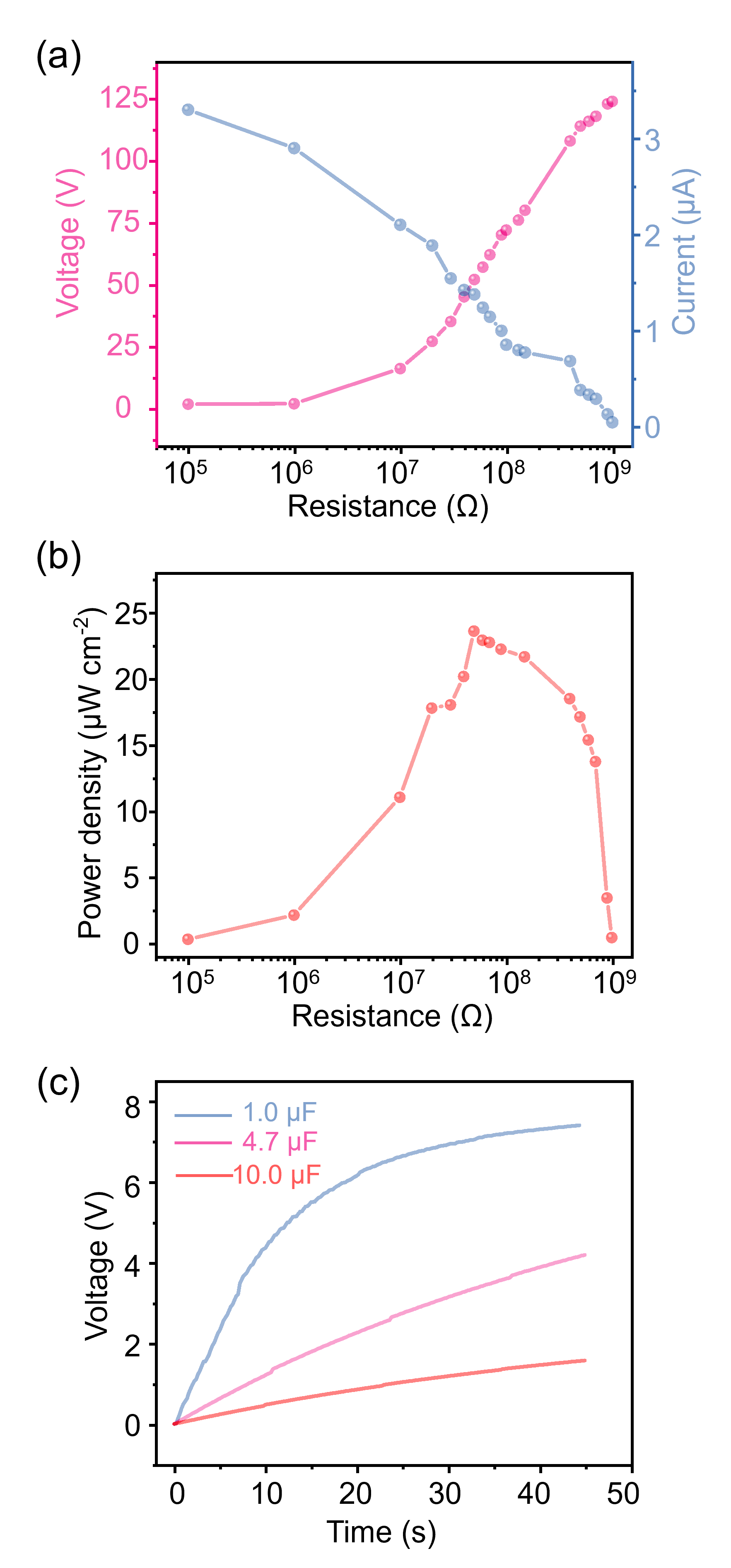


**Figure S18.** a) Voltage, current, and b) power density output characteristics of bGaIn-based TENG on paper for the external load resistance from 10^5^ to 10^9^ Ω. c) Demonstration of the energy harvesting from the TENG to charge capacitors of 1, 4.7, and 10 µF.


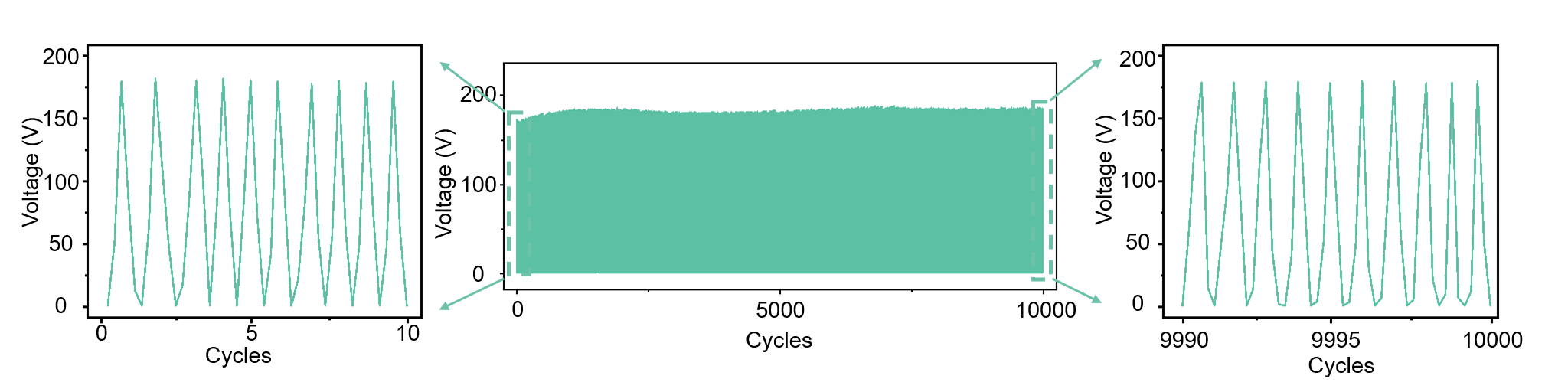


**Figure S19.** Stability test of the bGaIn-based TENG on paper (2.0 cm $\times$ 2.0 cm) over 10,000 load cycles.


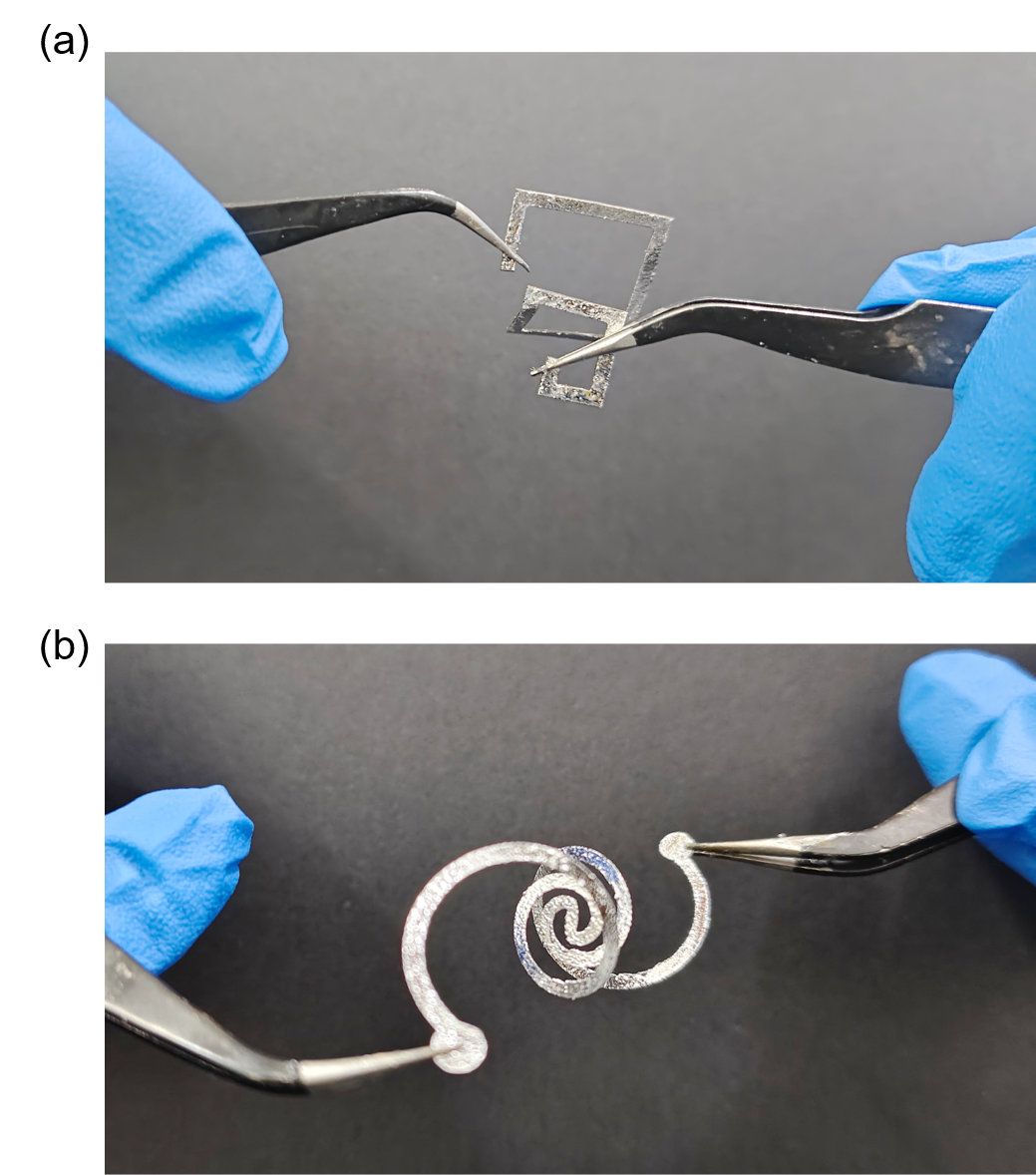


**Figure S20.** Stretchable bGaIn-based electrodes with a) spiral and b) ring shapes to accommodate the stretching deformation.


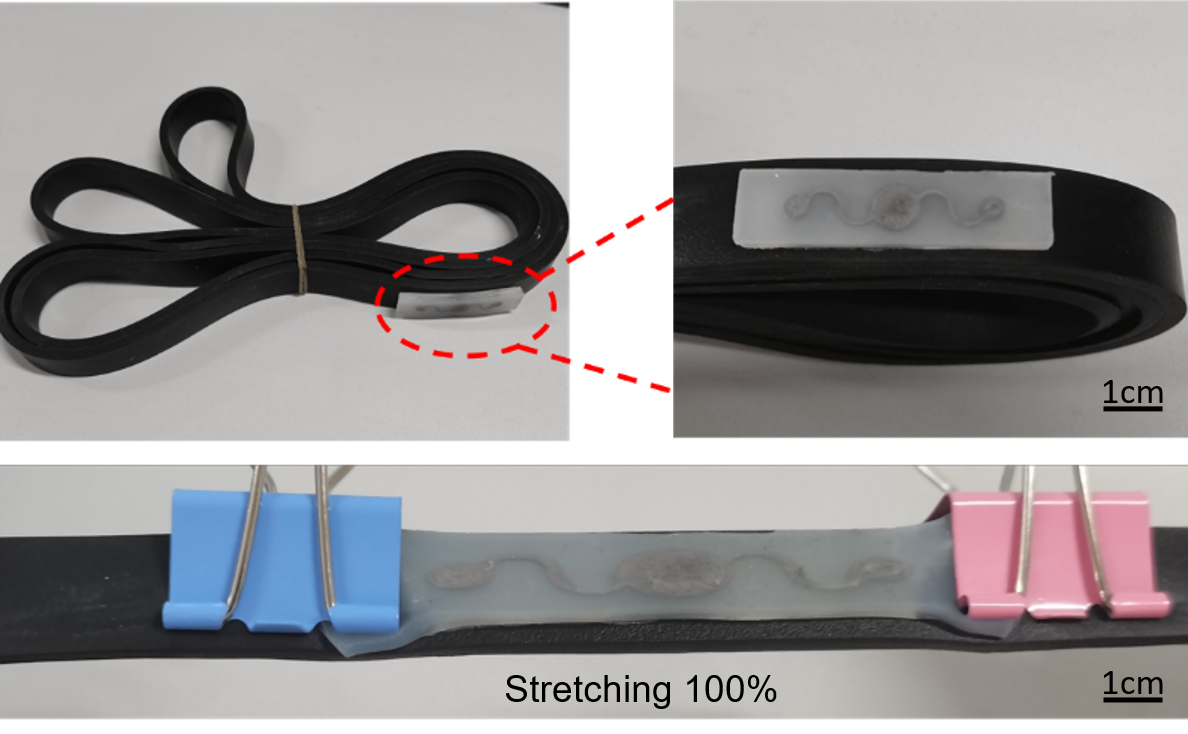


**Figure S21.** The smart elastic band integrated with stretchable TENG.


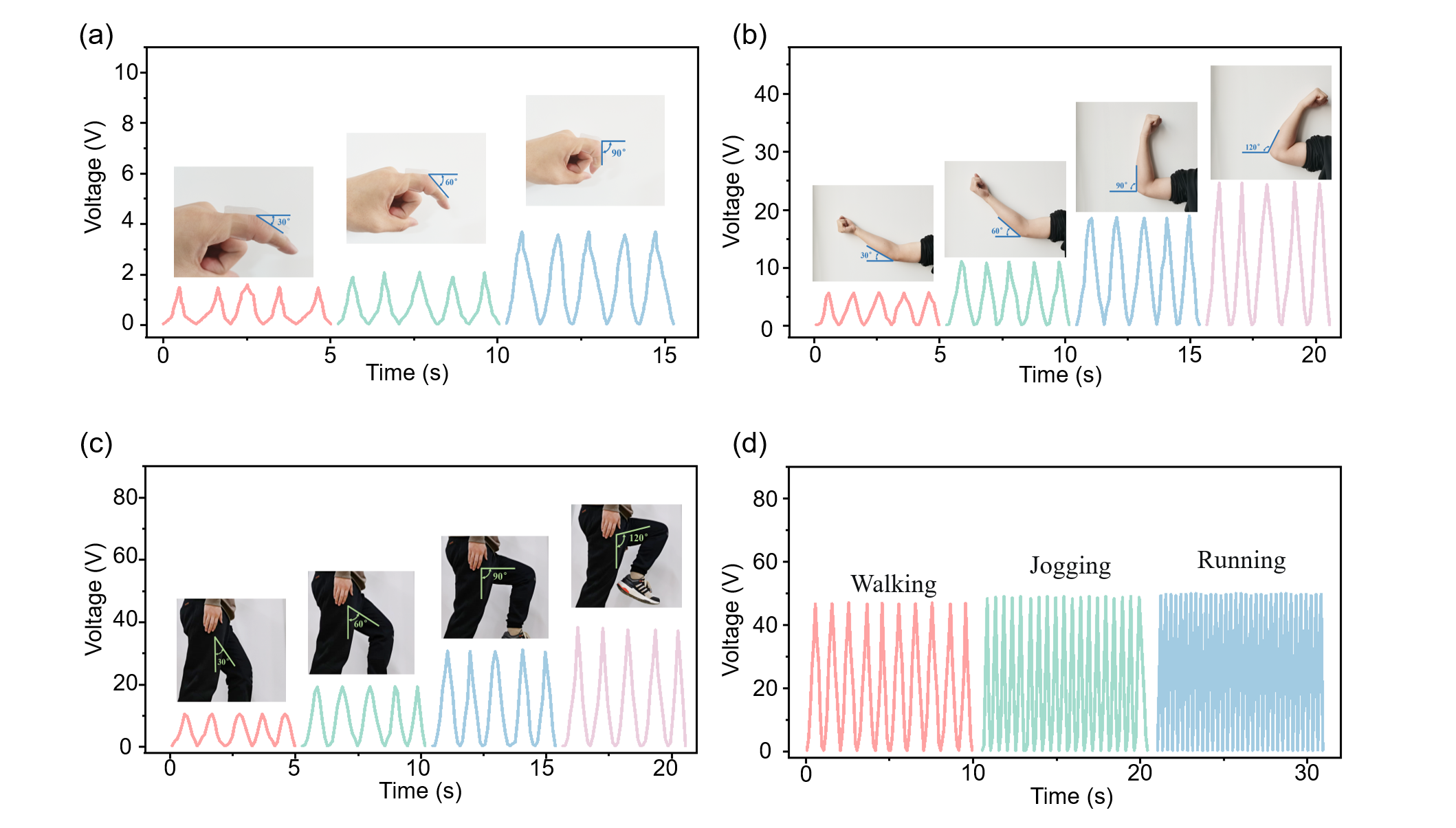


**Figure S22.** Monitoring of physical exercise and human motion with the EGaIn-based TENG: a) finger joint flexion, b) elbow and c) knee flexion angle, along with d) running, jogging, and walking.


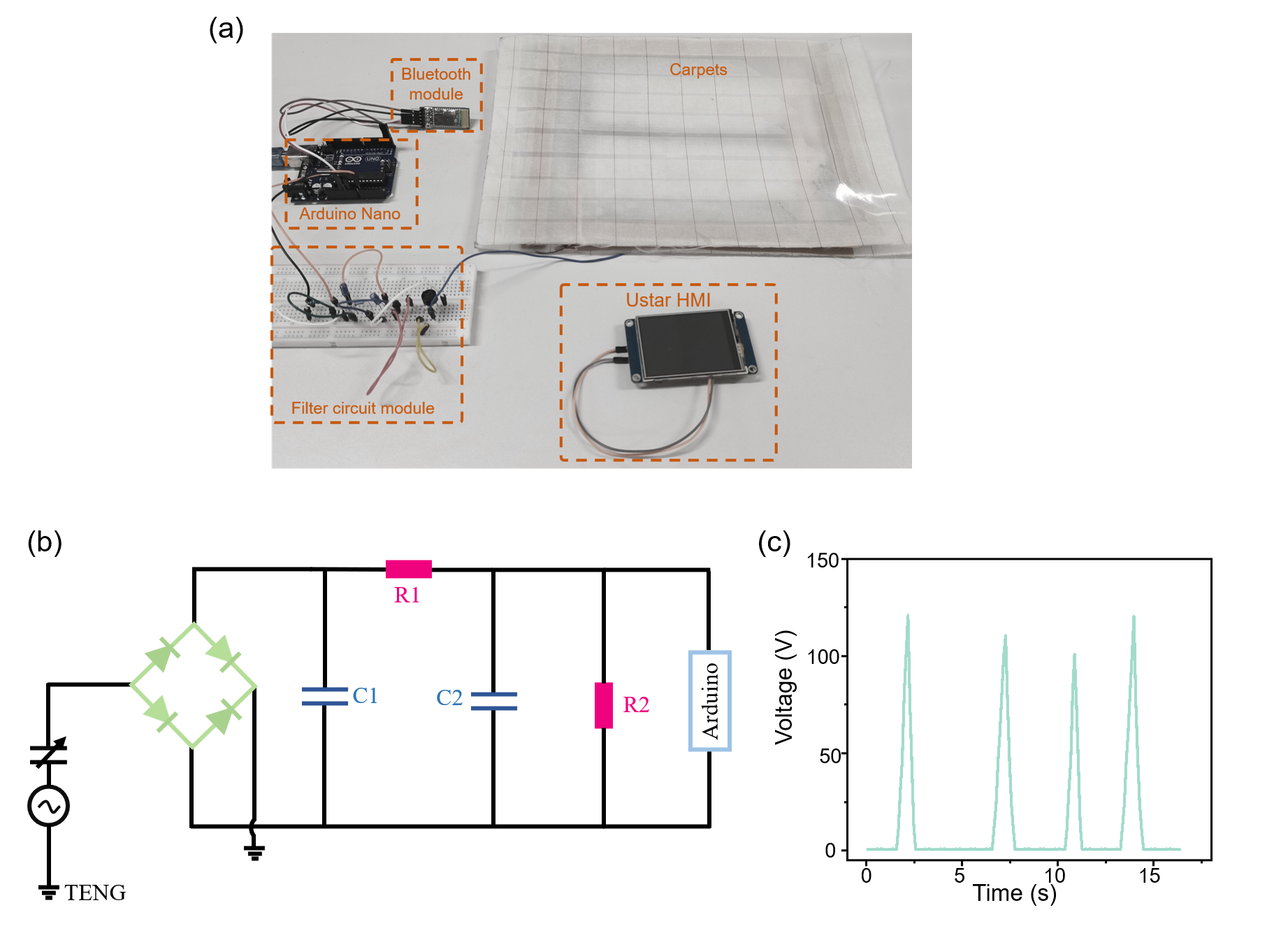


**Figure S23.** a) The optical image and b) circuit diagram of the wireless intelligent self-powered carpet, along with c) real-time voltage data from the smart carpet.


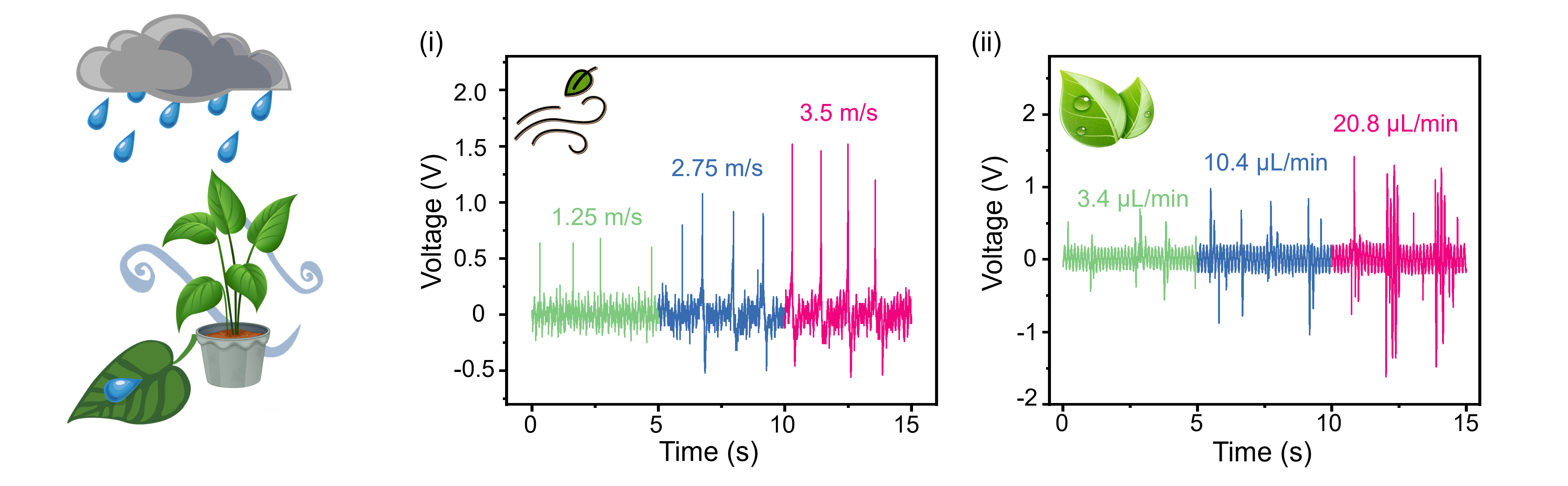


**Figure S24.** Application of the bGaIn-based TENG on the plant leaf to monitor wind speed and rainfall in natural environments.


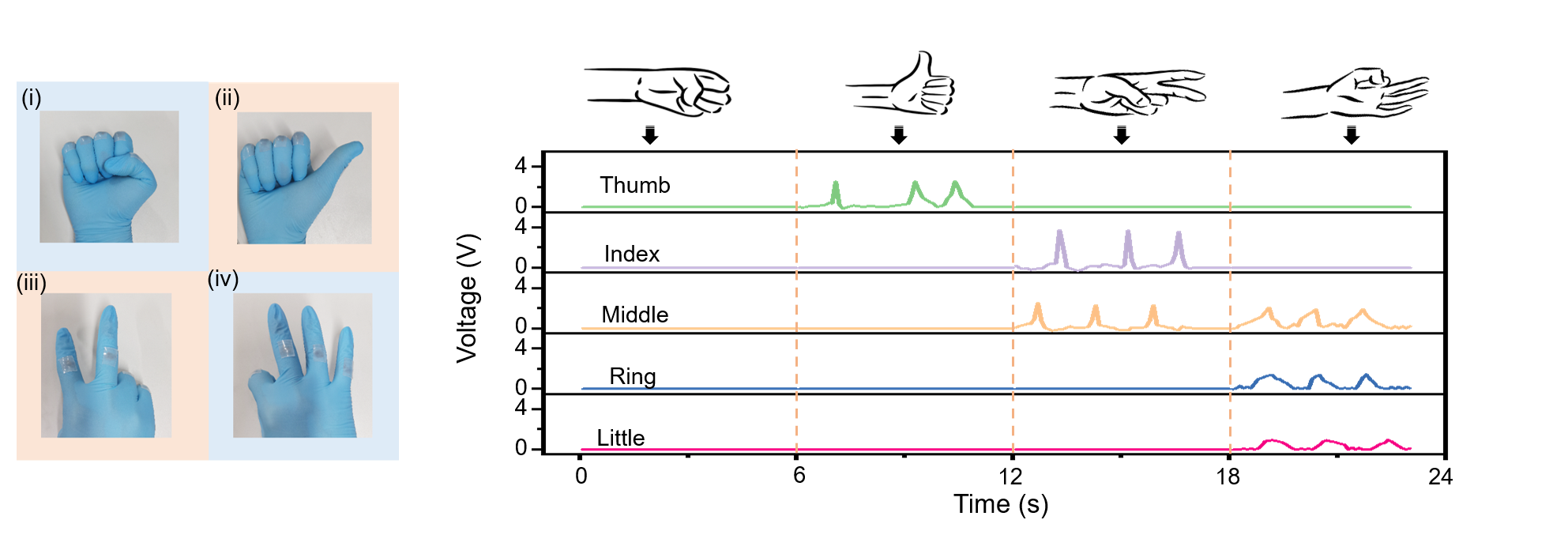


**Figure S25.** Gesture recognition with the bGaIn-based TENG on the glove.


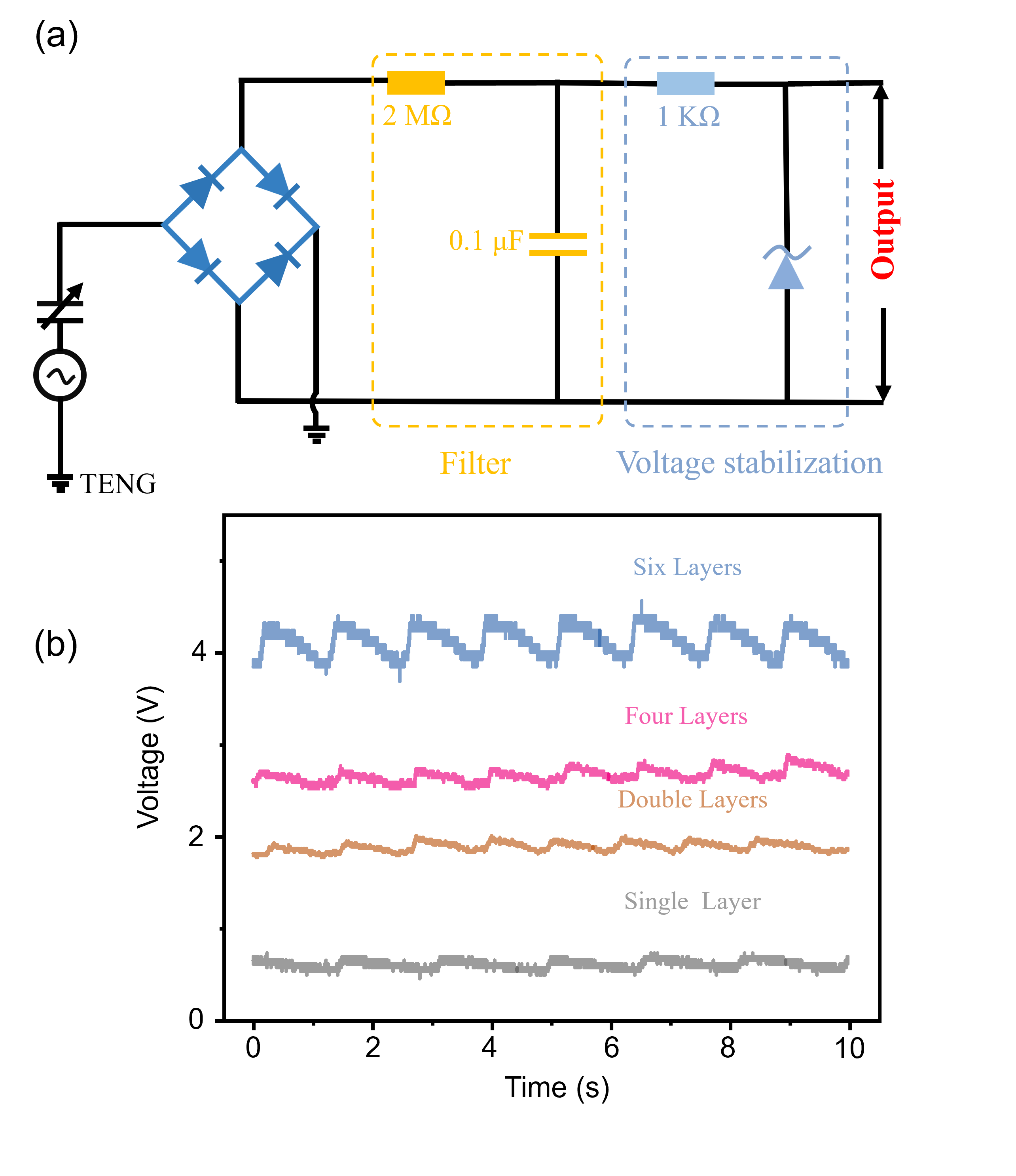


**Figure S26.** a) Diagram showing the voltage stabilization circuit. b) The bGaIn-based foldable TENG (with 1, 2, 4, and 6 layers) on paper for regulating voltage.


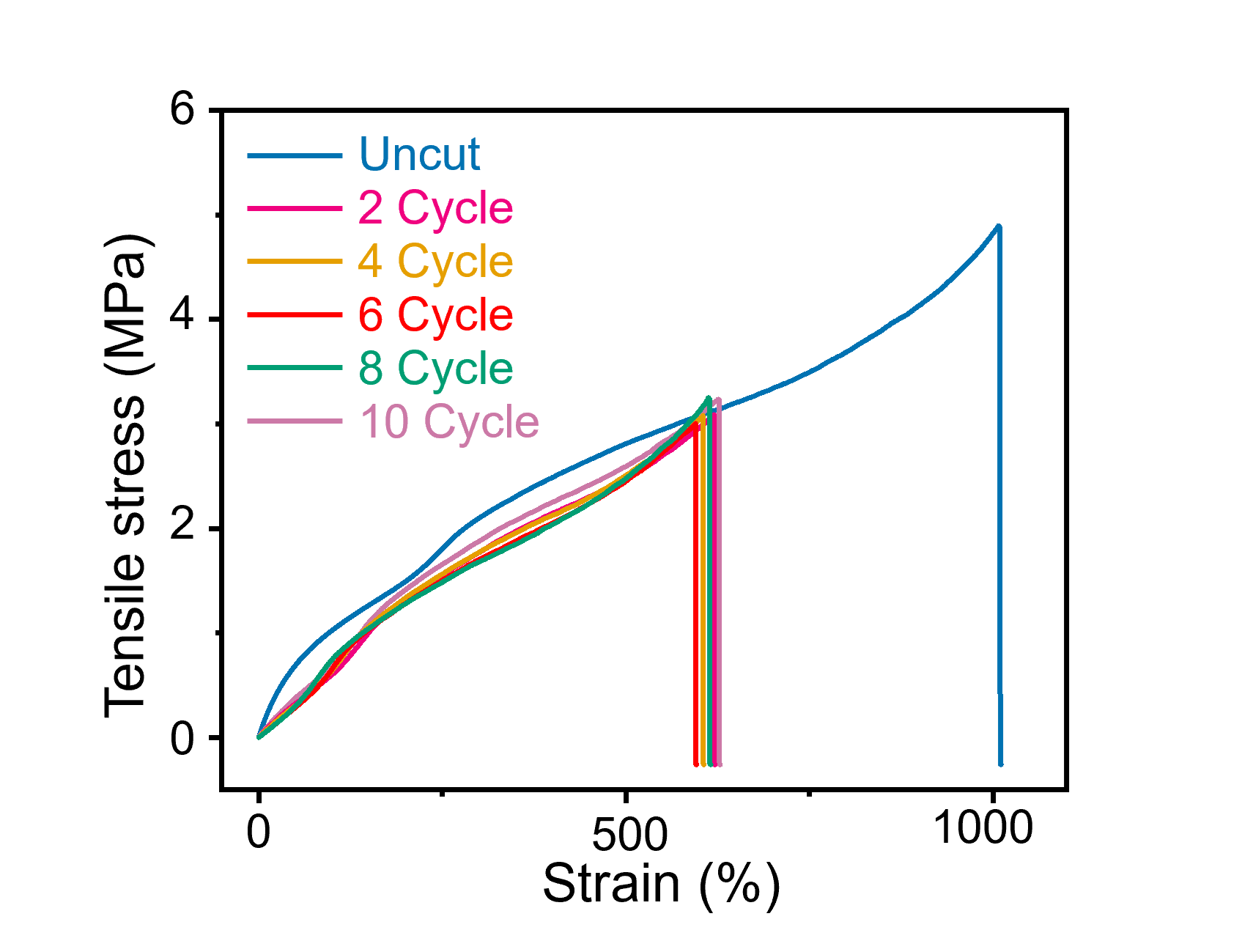


**Figure S27.** The stress-strain curves of the self-healing polymer over 10 cutting-healing cycles.


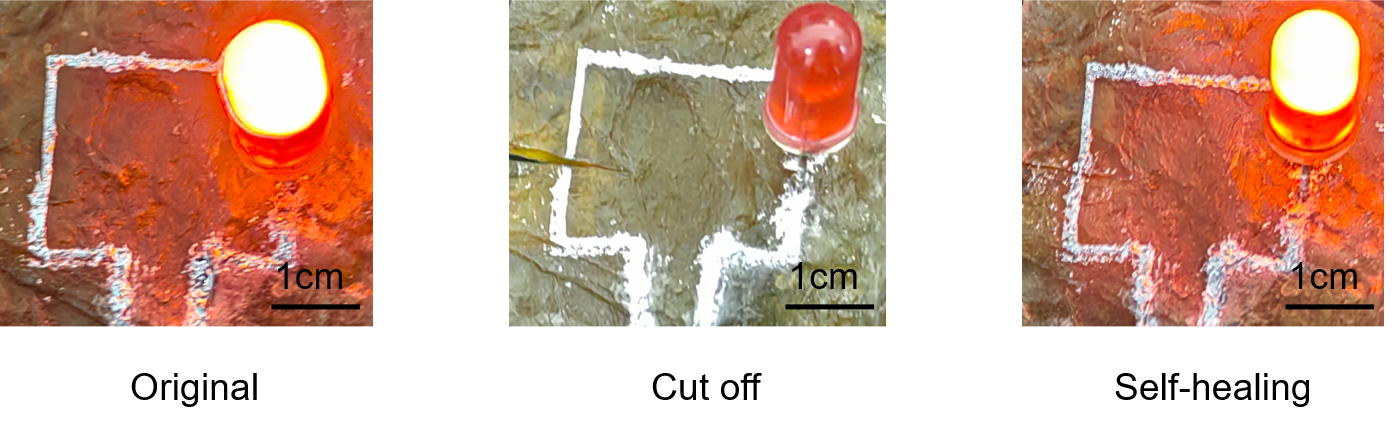


**Figure S28.** Changes in the brightness of the LED light: intact (left), damaged (middle), and self-healed (right) states.


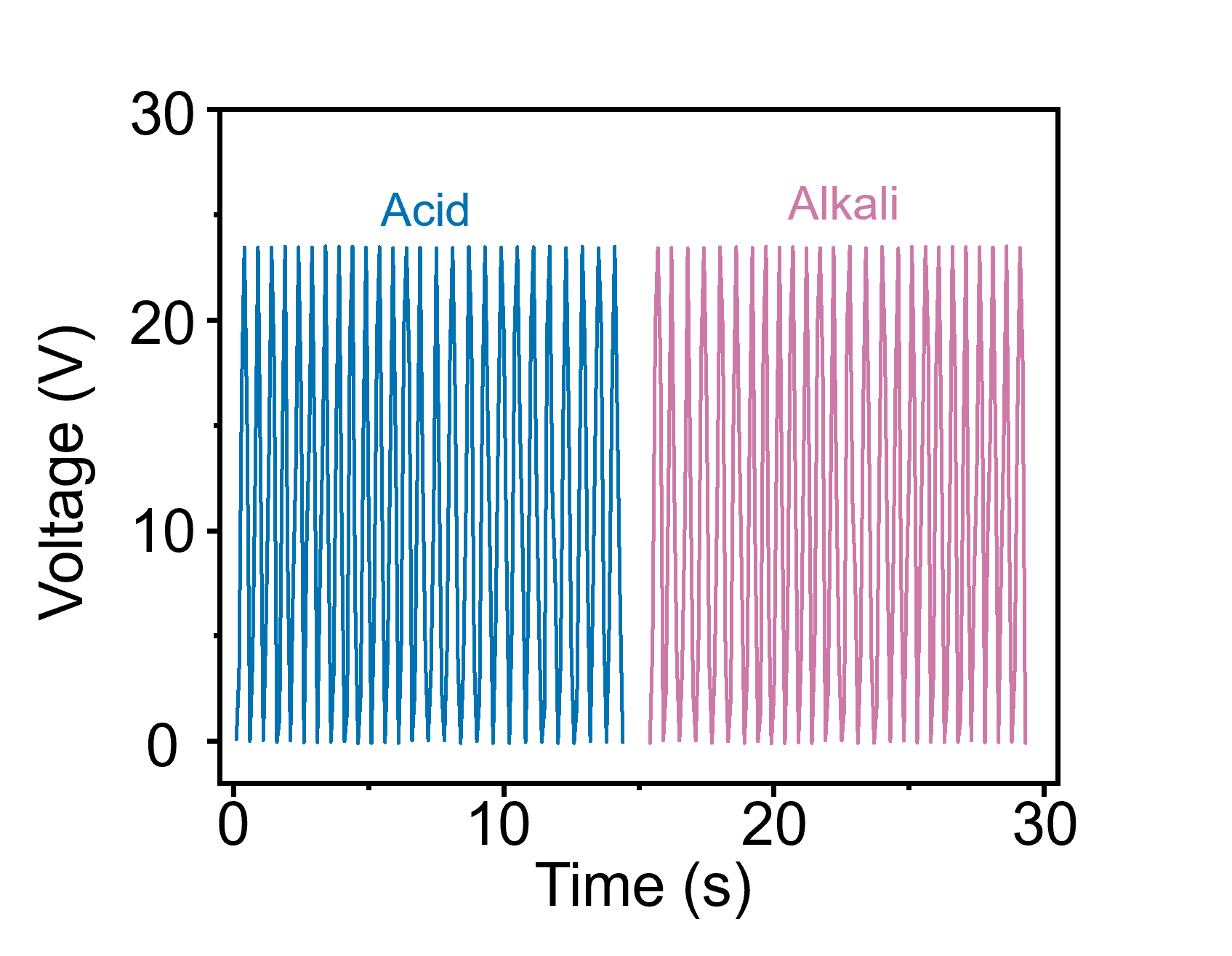


**Figure S29.** The electrical output characteristics after immersion of the device in acid and alkali solutions over 24 h.


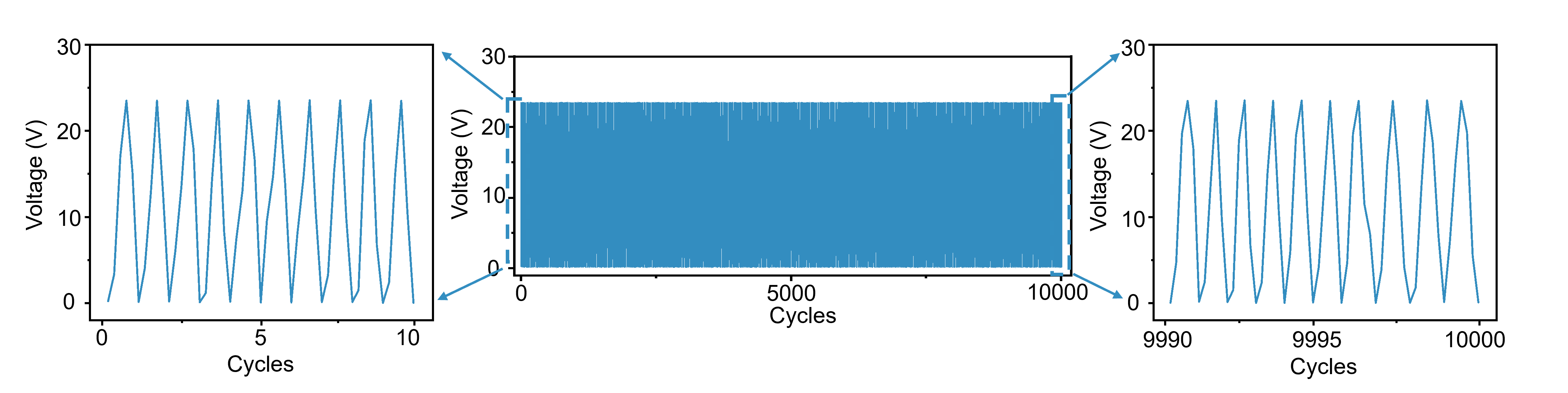


**Figure S30.** Stability test of the bGaIn-based TENG on self-healing polymers over 10,000 load cycles.


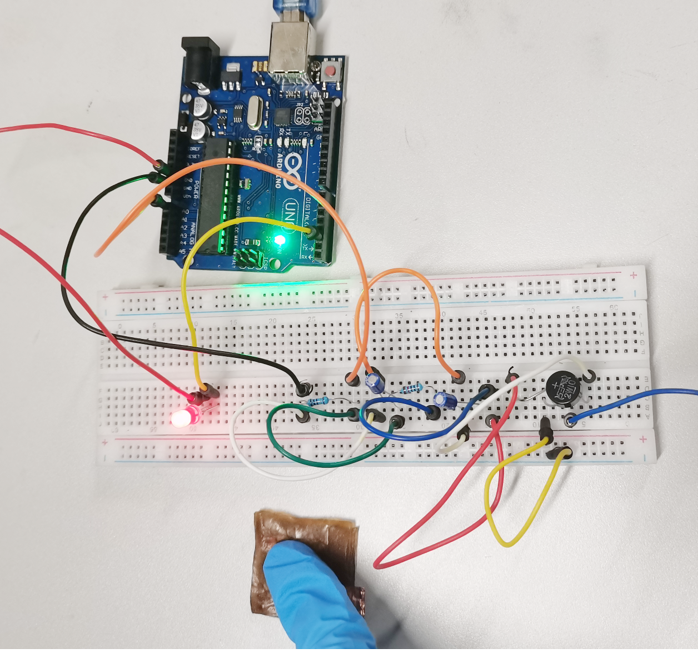


**Figure S31.** The optical image of the alarm system with the LED indicator driven by the TENG by hand clapping after acid splash.

**Table S1.** Performance comparison between stretchable TENGs in the literature report and this work.

| **Refs.** | **Size [cm^-2^]** | **V_OC_ [Vcm^-2^]** | | **I_SC_ [μAcm^-2^]** | **Power [μWcm^-2^]** | **Friction material** | **Conductive Electrode** | **Stretchability** |
| --- | --- | --- | --- | --- | --- | --- | --- | --- |
| **This work** | 5$\times$1.5 | 10.8417 | 0.1436 | | 7.5507 | Ecoflex 00-50&Nylon | EGaln | 867% |
| [1] | 5$\times$1.5 | 5.2000 | 0.0933 | | 2.0000 | Ecoflex 00-10&Skin | EGaIn | 140% |
| [2] | 8$\times$4 | 9.3750 | 0.1719 | | 50.4900 | Ecoflex 00-30&Skin | MXene/CNFs | 120% |
| [3] | 3$\times$3 | 31.6667 | 1.7200 | | 6.2600 | Ecoflex 00-10&Skin | Hydrogel | 150% |
| [4] | 5$\times$5 | 1.4288 | 0.2040 | | 7.2868 | SSG\PDMS&skin | GaInSn | 187% |
| [5] | 4$\times$8 | 9.9063 | 0.5625 | | 32.2000 | Ecoflex 00-30&Skin | LiCL/GO/EG | 200% |
| [6] | 5$\times$5 | 1.6000 | 0.0550 | | 4.0000 | Silicone&Nitrile glove | Gel | 200% |
| [7] | 2$\times$5 | 23.0000 | 0.0270 | | 33.0000 | Ecoflex 00-50&Kapton | MXene/PVA | 200% |
| [8] | 3$\times$3 | 8.8889 | 0.2222 | | 6.7900 | VHB&PU | lonogel | 200% |
| [9] | 6$\times$3 | 19.6389 | 0.8667 | | 0.8430 | Ecoflex 00-30&skin | GaInSn | 300% |
| [10] | 3$\times$6 | 14.7220 | 1.3830 | | 1.3800 | Ecoflex 00-30&Skin | PEDOT:PSS | 300% |

**Table S2.** Output characteristics of TENG normalized by weight.

| **Type** | **Height (mm)** | **Weight (g)** | **Voltage/Weight (v/g)** |
| --- | --- | --- | --- |
| Sretchable TENG | 4 | 2.7116 | 24.98 |
| bGaIn-based TENGs on the PET substrate | 0.632 | 0.6258 | 76.96 |
| bGaIn-based TENGs on the nylon substrate | 0.527 | 0.5322 | 96.26 |
| bGaIn-based TENGs on the leaf substrate | 0.965 | 0.7143 | 81.34 |
| bGaIn-based TENGs on the rubber substrate | 0.723 | 0.5476 | 133.67 |
| bGaIn-based TENGs on the self-healing polymer substrate | 2.004 | 0.7210 | 135.53 |
| bGaIn-based TENGs on the paper substrate | 0.351 | 0.4869 | 216.23 |

| **Refs.** | **V_OC_ [Vcm^-2^]** | **Power [μWcm^-2^]** | **Friction material** | **Conductive Electrode** | **Liquid metals treatment method** | |
| --- | --- | --- | --- | --- | --- | --- |
| **This work** | 44.8 | 23.6 | Ecoflex 00-50&Nylon | bGaln | phase change |  |
| [1] | 5.2000 | 2.0000 | Ecoflex 00-10&Skin | EGaIn | encapsulate |  |
| [11] | 40 | 21.966 | PHS&Skin | LM/Ag flskes/SEBS | dopant |  |
| [12] | 18.6 | 4.22 | SPSM&Skin | LM/AgFKs/SIS | sputter deposition |  |
| [13] | 19.75 | 9.52 | MPLC&Skin | MPLC | dip-coating |  |
| [14] | 22.9 | 55.16 | SSE&Al | Liquid metal | unprocessed |  |

**Table S3.** Performance comparison between TENGs based on bGaIn electrode in the literature report and this work.

**Table S4.** Comparison of bGaIn-based TENG with different electrode shapes.

| **Electrode shape** | **Electrode area (mm^2^)** | **bGaIn (g)** | **Device weight(g)** |
| --- | --- | --- | --- |
| Block | 250.00 | 0.1263 | 0.5563 |
| Petal | 236.94 | 0.1203 | 0.5503 |
| Spiral | 208.80 | 0.1076 | 0.4453 |
| Ring | 206.00 | 0.1064 | 0.4269 |
| Stripe | 204.00 | 0.1053 | 0.4159 |

**References:**

1. Y. Wu, Y. Li, Y. Zou, W. Rao, Y. Gai, J. Xue, L. Wu, X. Qu, Y. Liu, G. Xu, L. Xu, Z. Liu, Z. Li, *Nano Energy* **2022**, *92*, 106715.
2. W. T. Cao, H. Ouyang, W. Xin, S. Chao, C. Ma, Z. Li, F. Chen, M. G. Ma, *Adv. Funct. Mater.* **2020**, *30*, 2004181.
3. D. Bao, Z. Wen, J. Shi, L. Xie, H. Jiang, J. Jiang, Y. Yang, W. Liao, X. Sun, *J. Mater. Chem. A* **2020**, *8*, 13787.
4. S. Wang, L. Ding, X. Fan, W. Jiang, X. Gong, *Nano Energy* **2018**, *53*, 863.
5. X. Zhao, Z. Wang, Z. Liu, S. Yao, J. Zhang, Z. Zhang, T. Huang, L. Zheng, Z. L. Wang, L. Li, *Nano Energy* **2022**, *96*, 107067.
6. T. Jing, B. Xu, Y. Yang, *Nano Energy* **2021**, *84*, 105867.
7. X. Luo, L. Zhu, Y. C. Wang, J. Li, J. Nie, Z. L. Wang, *Adv. Funct. Mater.* **2021**, *31*, 2104928.
8. C. Dang, C. Shao, H. Liu, Y. Chen, H. Qi, *Nano Energy* **2021**, *90*, 106619.
9. Y. Yang, N. Sun, Z. Wen, P. Cheng, H. Zheng, H. Shao, Y. Xia, C. Chen, H. Lan, X. Xie, C. Zhou, J. Zhong, X. Sun, S. T. Lee, *ACS Nano* **2018**, *12*, 2027.
10. J. Shi, X. Chen, G. Li, N. Sun, H. Jiang, D. Bao, L. Xie, M. Peng, Y. Liu, Z. Wen, X. Sun, *Nanoscale* **2019**, *11*, 7513.
11. Y. Li, J. Xiong, J. Lv, J. Chen, D. Gao, X. Zhang, P. Lee, *Nano Energy*, **2020**, 78, 105358.
12. Y. Li, S. Xiao, X. Zhang, P. Jia, S. Tian, C. Pan, F. Zeng, D. Chen, Y. Chen, J. Tang, J. Xiong, *Nano Energy*, **2022**, 98, 107347.
13. X. Yang, C. Xu, K. Feng, L. Yang, S. Yin, *Composites Communications*, **2023**, 40, 101602.
14. L. Gong, T. Xuan, S. Wang, H. Du, W. Li, *Nano Energy*, **2023**, 109, 108280.
